# Is What Comes out the Same as What Goes in? A Preliminary Investigation of the Isotopic Impacts of Digestion by Red‐Tailed Hawks ( *Buteo jamaicensis* ) and Eurasian Eagle Owls ( *Bubo bubo* )

**DOI:** 10.1002/ece3.71211

**Published:** 2025-05-26

**Authors:** Brooke Erin Crowley, Madison Laurel Greenwood, Rachel Elizabeth Brown Reid

**Affiliations:** ^1^ Department of Geosciences University of Cincinnati Cincinnati Ohio USA; ^2^ Department of Anthropology University of Cincinnati Cincinnati Ohio USA; ^3^ Department of Veterinary Technology University of Cincinnati Blue Ash Blue Ash Ohio USA; ^4^ Department of Geosciences Virginia Tech Blacksburg Virginia USA

**Keywords:** Accipitridae, carbon isotopes, nitrogen isotopes, oxygen isotopes, Strigidae, strontium isotopes

## Abstract

We investigated isotopic diet‐excreta offset (Δ_diet‐excreta_) for predatory birds, and the isotopic influence of bird digestion on consumed prey tissues. Foraging ecology of predatory birds can be non‐invasively monitored using excreta or regurgitated prey. However, one must account for Δ_diet‐excreta_ and any influence of digestion on prey tissues. Neither of these has been previously evaluated for predatory birds. We worked with a captive Eurasian eagle owl ( *Bubo bubo* ) and red‐tailed hawk ( *Buteo jamaicensis* ) fed frozen murid rats. We collected rat feet, as well as regurgitated pellets and excreta from each bird's enclosure. We analyzed carbon (δ^13^C) and nitrogen (δ^15^N) isotopes in undigested rat muscle, undigested and digested fur, and bone collagen (extracted from pellets), δ^13^C, oxygen (δ^18^O), and strontium (^87^Sr/^86^Sr) isotopes in rat bone bioapatite, and δ^13^C, δ^15^N, and ^87^Sr/^86^Sr in bird excreta. Diet‐excreta offset differed slightly between individuals and depended on how we estimated diet (muscle alone or muscle + collagen) and if excreta were acidified. We tentatively suggest using +1 to 1.5‰ for Δ^13^C_diet‐excreta_ and +0.5 to 1‰ for Δ^15^N_diet‐excreta_ when working with proteinaceous tissues and unacidified or acidified excreta, respectively. For bioapatite, we suggest +9 to 10‰ for Δ^13^C_diet‐excreta_ and +0.001 for ^87^Sr/^86^Sr_diet‐excreta_. Fur isotopes, collagen δ^15^N, and bioapatite δ^18^O were unaffected by digestion, but ^87^Sr/^86^Sr decreased by 0.0005 to 0.001, and collagen and bioapatite δ^13^C shifted 0.5 to 1‰ (decreasing collagen‐apatite spacing by 1.5 to 2‰). For both birds, Δ_diet‐excreta_ for carbon and strontium were similar to previous studies, but nitrogen was notably different, possibly because excreta contained some urine or urates, or because raptors have distinct digestive physiologies. The influence of digestion on bone δ^13^C and ^87^Sr/^86^Sr is large enough to affect interpretations of diet and bioavailable strontium. Researchers should use caution if relying on potentially digested bone to evaluate the diet of consumed prey, establish strontium baselines, or infer past climate or environmental conditions.

## Graphical abstract

Isotope analysis of predator feces and consumed prey remains can be used to non‐invasively track diet and reconstruct past environment and climate conditions. However, one must account for the isotopic difference between diet and feces, as well as the possible influence of digestion on consumed prey tissues. We investigated the influence of digestion by a red‐tailed hawk (Rhett) and an eagle owl (Caspian) on murid rat tissues. We also investigated the isotopic offset between diet and feces for the two birds. We provide tentative correction values that can be used to account for diet‐feces offset for birds of prey and suggest researchers be cautious of using potentially digested bone to evaluate the diet of consumed prey or infer past climate or environmental conditions.

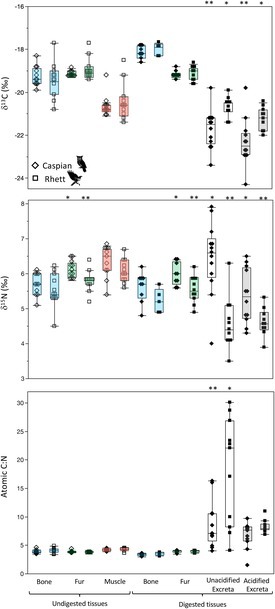

## 1 Introduction

The utility of non‐invasive methods for monitoring animal foraging ecology has been increasingly recognized over the past several decades. Among the methods that have emerged, fecal stable isotope analysis (specifically for carbon, δ^13^C, and nitrogen, δ^15^N) has been especially promising, and is now widely used to monitor an individual's diet or habitat use (reviewed in Reid et al. 2023). Because feces are a mix of undigested food and sloughed gut lining, their isotopic composition does not directly reflect that of diet. In order to be able to estimate the isotopic composition of diet based on feces, one needs to account for the isotopic offset, “Δ”, between diet and feces (e.g., Δ^13^C_diet‐feces_ = δ^13^C_diet_—δ^13^C_feces_). This has been extensively investigated for carbon and nitrogen in ungulates, as well as a few other mammals, but very little attention has been paid to other groups; to our knowledge, only two studies have investigated Δ_diet‐feces_ for invertebrates (Gorokhova and Hansson 1999, Deniro and Epstein 1978), one for reptiles (Steinitz et al. 2016), and this has not been studied at all for fish. Six studies have evaluated Δ_diet‐feces_ for birds (Tsahar et al. 2008; Mizutani and Wada 1988; Hatch 2012; Bird et al. 2008; Kuwae et al. 2022; Varo and Amat 2008), but this research has been limited to water birds and songbirds (Table 1). Additionally, just three studies have previously evaluated diet‐feces offset for strontium (^87^Sr/^86^Sr), all for mammals (Weber et al. 2020; Lewis et al. 2017; Crowley et al. 2019).

**TABLE 1:** Published diet‐excreta offset for birds.

Common name	Species	Diet	*N* individuals analyzed	Mean Δ^13^C_diet‐excreta_ ± 1σ (‰)	Mean Δ^15^N_diet‐excreta_ ± 1σ (‰)	Sample pre‐treatment	Source	Notes
Red‐necked stint	*Calidris ruficollis*	Cereal‐based pellets	3	0.2 ± 0.5	0.6 ± 0.6	Soaked in 1M HCl and homogenized	Kuwae et al. (2022)	Individuals kept in a shared enclosure and fed this diet for two days; 77 samples analyzed.
Red‐necked stint	*Calidris ruficollis*	Fish‐based pellets	3	−0.1 ± 0.3	0.5 ± 0.5	Soaked in 1M HCl and homogenized	Kuwae et al. (2022)	Individuals kept in a shared enclosure and fed this diet for two days; 20 samples analyzed.
Canada Goose	*Branta canadensis*	Adequate protein	4^a^	1.4 ± 1.1	—	Homogenized	Hatch (2012)	Excreta collected from goslings (age 14–21 to 74 days) kept in individual cages. Data extracted using automeris.io
Canada Goose	*Branta canadensis*	Deficient protein	3^a^	−0.3 ± 0.4	—	Homogenized	Hatch (2012)	"
Snow Goose	*Anser caerulescens*	Adequate protein	3^a^	1.3 ± 0.2	—	Homogenized	Hatch (2012)	"
Snow Goose	*Anser caerulescens*	Deficient protein	3^a^	1.5 ± 0.2	—	Homogenized	Hatch (2012)	"
Common coot	*Fulica atra*	Commercial bird food	16	0.7 ± 0.9	−2.6 ± 1.7	Homogenized	Varo and Amat (2008)	Excreta collected from aviary floor after birds fed diet for two days in Autumn
Common coot	*Fulica atra*	Commercial bird food	16	1.0 ± 0.9	−2.4 ± 0.9	Homogenized	Varo and Amat (2008)	Excreta collected from aviary floor after birds fed diet for two days in Spring
Common coot	*Fulica atra*	Pondweed (*Potomogeton pectinatus*)	16	0.7 ± 1.1	−0.4 ± 0.3	Homogenized	Varo and Amat (2008)	Excreta collected from aviary floor after birds fed diet for two days in Autumn
Common coot	*Fulica atra*	Pondweed (*Potomogeton pectinatus*)	16	0.0 ± 0.3	0.0 ± 1.0	Homogenized	Varo and Amat (2008)	Excreta collected from aviary floor after birds fed diet for two days in Spring
Red knobbed coot	*Fulica cristata*	Commercial bird food	16	0.4 ± 0.8	−3.3 ± 1.3	Homogenized	Varo and Amat (2008)	Excreta collected from aviary floor after birds fed diet for two days in Autumn
Red knobbed coot	*Fulica cristata*	Commercial bird food	16	1.1 ± 0.9	−3.4 ± 1.1	Homogenized	Varo and Amat (2008)	Excreta collected from aviary floor after birds fed diet for two days in Spring
Red knobbed coot	*Fulica cristata*	Pondweed (*Potomogeton pectinatus*)	16	0.2 ± 0.5	−1.2 ± 0.4	Homogenized	Varo and Amat (2008)	Excreta collected from aviary floor after birds fed diet for two days in Autumn
Red knobbed coot	*Fulica cristata*	Pondweed (*Potomogeton pectinatus*)	16	0.0 ± 0.2	−4.5 ± 4.2	Homogenized	Varo and Amat (2008)	Excreta collected from aviary floor after birds fed diet for two days in Spring
Adelie Penguin	*Pygoscelis adeliae*	Natural diet (assume fish)	Unknown	1.7	−1.0 ± 0.4	Preserved in ethanol in the field; dried, homogenized, and passed through a 0.5 mm sieve	Mizutani and Wada (1988)	Four fresh droppings collected from the ground in a wild bird rookery. Diet estimated by the authors based on measured values for possible foods and expected diet‐tissue fractionation for feathers.
Black‐tailed Gull	*Larus crassirostris*	Natural (assume fish and crustaceans)	Unknown	−0.3	−0.3	Stored frozen and then dried and homogenized	Mizutani and Wada (1988)	"
Yellow‐vented bulbul	*Pyconotus xanthopygos*	Low casein protein diet	5	—	−0.9 ± 0.2	Homogenized	Tsahar et al. (2008)	Birds housed individually and fed a "standard maintenance" banana and soy protein mash for 75 days followd by a prescribed protein diet for 95 days. Excreta collected from sheets lining each individual's enclosure on the day prior to the dieta switch (day 0) and then on days 2, 4, 9, 21, 35, 57, 80 and 95 of consuming prescribed diet.
Yellow‐vented bulbul	*Pyconotus xanthopygos*	Medium protein diet	4	—	−0.8 ± 0.3	Homogenized	Tsahar et al. (2008)	"
Yellow‐vented bulbul	*Pyconotus xanthopygos*	High protein diet	4	—	−0.6 ± 0.3	Homogenized	Tsahar et al. (2008)	"
Zebra finch	*Taeniopygia castanotis*	Zebra finch mix (five types of millet and canary seed)	12	−0.3 ± 0.5	−1.1 ± 0.2	Homogenized	Bird et al. (2008)	Excreta collected from sheets lining shared enclosures. Birds fed this diet for the first 24 days of the study. Reported summary data are for samples collected on days 7, 14, 21 and 24
Zebra finch	*Taeniopygia castanotis*	Canary seed only	12	0.1 ± 0.3	−0.1 ± 0.3	Homogenized	Bird et al. (2008)	Excreta collected from sheets lining shared enclosures. Birds fed this diet after 24 days. Reported summary data are for samples collected on days 28, 29, 30, 32, 36, and 39

*Note:* With the exception of the snow goose and Canada goose samples included in Hatch (2012), which had minimal uric acid coating, all reported offsets are for bulk dried excreta rather than feces.

^a^ Number of individuals analyzed and number of samples collected not explicitly stated in the publication. Number of individuals inferred based on figures for other tissues and assumed that a single sample analyzed from each individual.

While fecal material alone can provide important information about diet and behavior, undigested remains of consumed prey can also be incredibly informative (reviewed in Reid et al. 2023). Under the right conditions (e.g., within rock shelters and cave entrances), feces can accumulate and preserve for decades to centuries, providing an archive of past climate and vegetation (e.g., Chase et al. 2012; Cleary et al. 2018). However, under most conditions, fecal matter breaks down quickly. For predatory species, it may alternatively be possible to non‐invasively obtain ecological information by analyzing isotope values in remains of consumed prey (Reid and Koch 2017; Porder et al. 2003; Crowley et al. 2019, 2017). Bones, teeth, fur, feathers, tests, and shells of consumed (and regurgitated) organisms persist on the landscape much longer than fecal matter, and accumulations of prey remains can provide an archive of long‐term behavioral trends, as well as regional climate and environmental conditions. Researchers have used carbon, strontium, and oxygen (δ^18^O) isotope values in accumulated prey remains to establish environmental baselines, as well as track dietary intake and monitor landscape use for both predators and consumed prey (Crowley et al. 2017; Cooke and Crowley 2018; Copeland et al. 2008, 2010; Gehler et al. 2012; Leichliter et al. 2017; Janzen et al. 2020; Terry 2018). Osseous tissues are physically altered via microscopic, and sometimes macroscopic, digestive etching (Terry et al. 2018). However, we are not aware of research evaluating the degree to which digested and regurgitated remains, including bones and teeth, are isotopically altered.

To help address these knowledge gaps, we conducted a semi‐controlled feeding study with two captive predatory birds (hereafter referred to as raptors) from the Cincinnati Zoo: One adult female red‐tailed hawk ( *Buteo jamaicensis* ) called “Rhett”; and one adult female Eurasian eagle owl ( *Bubo bubo* ) called “Caspian”. We measured the isotopic composition of bone collagen, bone bioapatite, fur, and muscle from *Rattus* sp. fed to the birds before digestion, bone and fur of the rodents after digestion, and raptor excreta for a total of 11 days. We analyzed carbon and nitrogen isotopes for all proteinaceous materials, carbon, oxygen, and strontium isotopes for bone bioapatite, and carbon, nitrogen, and strontium isotopes for feces.

### 1.1 Species Descriptions

Red‐tailed hawks are a widespread North American species of accipitrid raptor belonging to the order Accipitriformes. Females weigh between 900 and 1460 g and have a wingspan of 114–133 cm (Preston 2000; Preston and Beane 2009). Eurasian eagle owls, which belong to the Strigidae family within the order Strigiformes, have a very broad distribution ranging from western Europe through east Asia (including Korea and Japan), south into the Arabian Peninsula, and India, and historically Northern Africa (Vaurie 1963; Holt et al. 2020). Eagle owls have a wingspan of 160–188 cm, and females range in body mass from 1.75 to 4.8 kg (Vaurie 1963; Holt et al. 2020; Penteriani and Del Mar Delgado 2019). Small mammals (particularly rodents) comprise the majority of both species' diets, but both red‐tailed hawks and eagle owls consume a wide range of prey, including birds, reptiles, fish, and invertebrates (Penteriani and Del Mar Delgado 2019; Preston 2000; Preston and Beane 2009; Holt et al. 2020).

Until recently, red‐tailed hawks and other accipitrids were placed within the Falconiformes order along with falcons. However, recent genetic analysis has indicated that falcons are phylogenetically distinct and more closely related to Psittaciformes (parrots), Passeriformes (songbirds), and Cariamiformes, a small order including extant seriamas and extinct terror birds (Prum et al. 2015; Cho et al. 2019). Falconiformes, Accipitriformes, and Strigiformes are thought to have diverged in the early Cenozoic (Cho et al. 2019), so there could be some real differences in digestive physiology among them. Most work on raptor physiology was conducted prior to the recent genetic work, and thus considers hawks to be within the Falconiformes and frequently summarizes results at the ordinal level without noting or highlighting possible distinctions or differences between accipitrids and falcons. However, in many instances, work was conducted on the *Buteo* genus specifically, and we have relied on those data when summarizing trends for red‐tailed hawks.

### 1.2 Raptor Digestive Physiology

Birds of prey have relatively short and simple gastrointestinal tracts compared to most mammals, which include an esophagus, a simple muscular stomach, a short intestine, and a colon (Houston and Duke 2007). For all raptors, digestion occurs primarily in the stomach, and nutrients are absorbed in the small intestine (Houston and Duke 2007). Most birds have a specialized portion of their esophagus, called a crop, or ingluvies, which helps mechanically break down food. However, because raptors primarily consume meat, which is relatively easy to digest, crops are poorly developed in accipitrids, and owls lack them entirely (Houston and Duke 2007; Pollock 2023). Conversely, accipitrids lack a cecum (Pollock 2023), but this organ has been retained in owls and may help with water retention, nutrient absorption, and breakdown of foods that require microbial assistance (Proszkowiec‐Weglarz 2022). Like reptiles, all birds, including hawks and owls, excrete waste through a single orifice called a cloaca. This waste is comprised of feces, liquid urine, and urate salts (Casotti and Braun 2004).

All raptors regurgitate a pellet composed of undigested material on a regular basis. The relative frequency of pellet production, and the amount of time that passes between when a meal is consumed and when a pellet is produced (called meal‐to‐pellet interval, or MPI) can vary depending on what was consumed, when it was consumed, and by whom (Duke et al. 1975, 1976). MPI for owls is mostly influenced by meal size (with bigger meals requiring more digestion time) while MPI for hawks is closely tied to photoperiod (birds tend to regurgitate pellets at dawn regardless of when they had their meal). Most birds regurgitate a single pellet following a meal, but this is not always the case. In some instances, individuals skip producing a pellet for a day, or produce multiple pellets a few hours apart after a single meal (Duke et al. 1975).

Red‐tailed hawks and eagle owls are expected to have similar digestive efficiency of meat. Both are “sit and wait” generalist ambush predators, which should have broadly similar intestinal digestive efficiency (Barton and Houston 1993). There are, however, major differences in the degree to which the two species digest bones. As anyone who has dissected an owl pellet can attest, owls digest relatively little bone and tend to regurgitate complete skeletal elements from their consumed prey. Accipitrids, on the other hand, produce pellets that contain very little bone. This is primarily due to differences in the pH of gastric juices among orders (2.3 for owls versus 1.6 for hawks), although there may also be small differences in proteolytic activity among orders and species (Duke et al. 1975). The tendency for owls to prefer meals that can be swallowed whole (Pollock 2023) may also help preserve delicate skeletal elements of small‐bodied prey.

### 1.3 Review of Diet‐Feces Offset

As noted above, Δ_diet‐feces_ has been most extensively studied for carbon and nitrogen isotopes in mammals (reviewed in Reid et al. 2023). Overall, this research suggests that fecal δ^13^C values tend to be lower than diet (resulting in positive Δ^13^C_diet‐feces_ values), while fecal δ^15^N values tend to be higher than diet (resulting in negative Δ^15^N_diet‐feces_ values), but there is a large amount of variability both within and among species, most likely due to variability in gut physiology, the nutritional composition of diet (including diet heterogeneity), and study design (reviewed in Reid et al. 2023). Diet‐feces offset has only been evaluated for four captive faunivorous mammal species fed semi‐controlled diets: meerkats ( *Suricata suricatta* ), tigers ( *Panthera tigris* ), snow leopards ( *Uncia uncia* ), and house cats ( *Felis catus* ) (Montanari and Amato 2015; Montanari 2017; Reid et al. 2023). Offsets for nitrogen isotopes were relatively comparable across species. They were smallest for omnivorous meerkats (−1.5 ± 1.1‰); tigers and house cats had very similar average Δ^15^N_diet‐feces_ (−1.6 ± 2.1‰ and −1.7 ± 0.6‰, respectively), while offset was larger for snow leopards fed the same diet as the tigers (−2.5 ± 1.5‰). In contrast, Δ^13^C_diet‐feces_ was highly variable among carnivorans (ranging from +1.4 ± 0.7‰ for house cats to −2.3 ± 3.6‰ for snow leopards).

Isotope analysis of avian excreta is relatively common, but only six studies have specifically investigated the offset between diet and excreta for birds (Table 1). We are using the term “excreta” rather than “feces” here because birds produce multiple waste products, including feces, uric acid, and urates (Crouch et al. 2020), and as Table 1 demonstrates, most researchers have not attempted to analyze these waste products separately. No work has been previously conducted with predatory birds. Given their high protein diet, we might expect raptors to have small Δ^13^C_diet‐excreta_ and Δ^15^N_diet‐excreta_ similar to other birds fed high protein diets. Alternatively, it is possible that they will look more like faunivorous mammals, which also have relatively simple digesta and short gut retention times but larger and more variable Δ^13^C_diet‐feces_ and Δ^15^N_diet‐feces_ (Reid et al. 2023; Stevens and Hume 2004).

Finally, to our knowledge, Δ_diet‐feces_ for strontium has only been previously evaluated in three studies (summarized in Appendix 1). Lewis et al. (2017) found negligible offset for pigs ( *Sus scrofa* ) fed diets with differing amounts of marine‐derived protein (ranging from −0.000004 to 0.000051) while Weber et al. (2020) found Δ^87^Sr/^86^Sr_diet‐feces_ ranged from ca. −0.0029 to +0.00043 for individual brown rats ( *Rattus norvegicus* ) and guinea pigs ( *Cavia porcellus* ) fed pellets composed of plants, insects, or vertebrate meat. Lastly, Crowley et al. (2019) estimated Δ^87^Sr/^86^Sr_diet‐feces_ for wild jaguars ( *Panthera onca* ) using undigested bone chunks removed from scats; offsets ranged from −0.00620 to +0.01797, with an average of 0.0022 ± 0.0067.

## 2 Methods

### 2.1 Sample Collection and Preparation

This study was conducted with permission from the Cincinnati Zoo, Ohio, USA. Rhett the hawk and Caspian the owl were individually housed and their enclosures were cleaned daily. Samples were collected by zoo staff during normal daily feeding and cleaning procedures from October 25 through October 28 in 2018, and July 3 through July 12 in 2019. This amounted to a total of 11 sampling days. Each day consisted of a 24‐h period. Both raptors were exclusively fed frozen lab rats ( *Rattus norvegicus* ) during the study. Zoo staff removed a whole foot from to‐be‐consumed rodents and then fed each bird the remaining carcasses. Staff then collected regurgitated pellets and a sample of dried excreta from each bird's enclosure on the following morning before serving the next day's meal. Collected samples were stored frozen at −18°C.

To track the isotopic influence of digestion, we analyzed: (1) undigested rodent bone, fur, and muscle; (2) bone and fur recovered from regurgitated pellets; and (3) excreta from both birds. Undigested rodent feet were thawed, dissected (separating fur, muscle, and bone), and air dried. Regurgitated pellets were freeze dried and then dissected (separating fur, claws, bone fragments, and teeth). Each separated tissue was then processed following previously established protocols (Crowley et al. 2010, 2019; Crowley and Wheatley 2014). Fur and muscle were repeatedly sonicated in trace metal grade petroleum ether to remove debris and lipids, rinsed several times with ultrapure water, and freeze dried overnight. Bone collagen (for C and N analysis) was isolated by demineralizing ~50 mg of small bone fragments in 0.5 N HCl at 4°C. Samples were rinsed with ultrapure water and then repeatedly sonicated at 10‐min intervals in petroleum ether to remove lipids. Each sample was sonicated four to 10 times. We removed liberated lipids and replaced petroleum ether after every rinse, and sonicated all samples one additional time after no lipids were visibly released to ensure complete removal of lipids. Samples were then sonicated two to three times with ultrapure water until the liquid was clear, and freeze dried overnight. Bone apatite (for C, O, and Sr analysis) was isolated by immersing roughly 20 mg of powdered pre‐ and post‐digested bone in 30% hydrogen peroxide for 72 h at room temp, rinsing with ultrapure water, soaking in 1 M acetic acid buffered with calcium acetate for 24 h at 4°C, rinsing again with ultrapure water, and freeze drying overnight. We note that because Rhett digested a fair amount of the bone from the rodents she consumed, there was not enough sample to analyze both bone collagen and bioapatite for all days of the study; we were only able to analyze bioapatite for four digested samples (see Appendix 2 for specific details about all samples, including raw isotope data).

Excreta were freeze dried prior to handling. We had hoped to be able to isolate the fecal portion of excreta for analysis. Unfortunately, this proved challenging, and we were not able to confidently separate feces from dried urine or urates in any of the samples. However, this challenge is not unusual to our study; as we reviewed above, all previous studies evaluating diet‐feces isotopic offsets for birds have used homogenized excreta (Table 1). We therefore refer to the samples as “excreta” throughout the remainder of the paper. Most of the samples for Rhett resembled tan mud smeared on the inside of the collection bags, although there were a few samples that were dark brown soil‐like crumbles mixed with the gravel used to line the floor of the bird's enclosure. Samples for Caspian varied in texture and included tan mud and dark brown crumbles similar to Rhett's excreta, dark brown to green semi‐transparent, treacle‐like shiny shards, and one odd sample (from 7/5/19) that was yellow‐gray and resembled elemental sulfur (see Appendix 2). Some samples contained multiple visibly distinct components, and we tried to separately sample these whenever possible for carbon and nitrogen analysis. Lastly, there were distinct white flecks in a few samples (*N* = 5 total; Appendix 2), which we thought might be likely candidates for urates (hereafter called “urate?”). We analyzed C and N isotopes for these as well.

**FIGURE 1:**
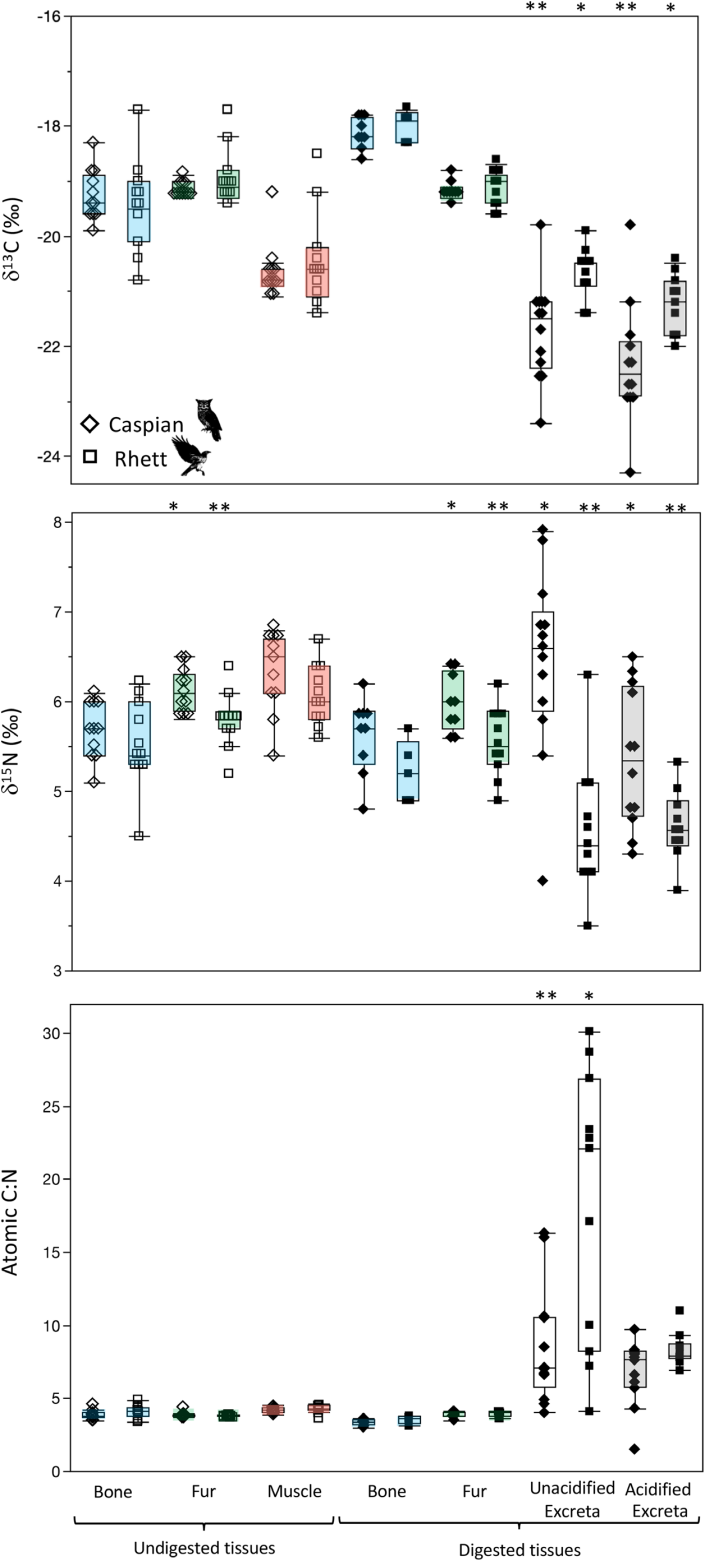
Box plots comparing δ^13^C, δ^15^N and atomic C:N for proteinaceous rodent tissues and raptor excreta for Caspian the eagle owl and Rhett the red‐tailed hawk. Asterisks denote comparisons that differ significantly between individual birds. Summary data and statistical comparisons among tissues for each individual are provided in Table 3. The single unacidified excreta with low δ^15^N for Caspian was from 10/26/18. Neither δ^13^C nor atomic C:N were unusual for this sample (21.2‰ and 4.9, respectively). The single acidified excreta with elevated δ^13^C for Caspian was from 7/4/19. Neither δ^15^N nor C:N were unusual for this sample (6.3‰ and 8.1, respectively). The anomalously low δ^13^C and small C:N data for an acidified excreta for Caspian were both from 7/5/19; δ^15^N for this sample was not unusual (6.1‰).

Some authors have advocated for acidifying excreta to remove carbonates and other minerals (Reid and Koch 2017; Kuwae et al. 2022). However, very few researchers follow this practice, possibly because there is mixed evidence for the necessity of this step, and the isotopic impact of soaking samples in acid is not well understood (and possibly contraindicated) (Schlacher and Connolly 2014). Unacidified raptor excreta had very large atomic C:N (Figure 1 and Appendix 2). We therefore decided it would be helpful to explicitly evaluate the influence of acid treatment on raptor excreta. With the exception of samples collected on 7/7/19 (which were small for both Rhett and Caspian), we had enough excreta material available to be able to acidify an aliquot of each sample. However, we were not able to chemically treat the white flecks noted above as they were rare and small. Samples were soaked in 0.5 N HCl for 5 h at room temperature in microcentrifuge tubes. We swapped out the acid solution at 2.5 h, and samples were frequently agitated, and lids were left loose to let evolved gas escape. At the end of 5 h, samples were rinsed five times with ultrapure water and freeze dried.

### 2.2 Sample Analysis

Carbon and nitrogen isotope ratios, as well as carbon and nitrogen content (weight %C and weight %N) in fur, muscle, bone collagen, and excreta were analyzed in the Stable Isotope Biogeochemistry Lab at the University of Cincinnati. Between 0.35 and 0.45 mg of material were weighed into tin cups and analyzed on a Costech Elemental Analyzer connected to a Thermo Scientific Delta V IRMS (Bremen, Germany) via a Costech Conflo IV interface (Valencia California, USA). Carbon and nitrogen isotope data are reported relative to the international scales Vienna Pee Dee Belemnite (VPDB) and atmospheric air (AIR). Following Skrzypek (2013), four reference materials were included in each run and used to correct data. We accounted for linearity and drift using powdered caffeine and did a two‐point normalization for scale using caffeine (−38.3‰ and 1.6‰ for carbon and nitrogen, respectively) and USGS 41 (37.6‰; 47.6‰). Accuracy for protein runs (based on three replicates of independent references powdered glycine and purified gelatin interspersed throughout each run) was 0.46‰ for carbon and 0.24‰ for nitrogen. Accuracy for fecal runs (based on glycine and soy flour) was 0.06‰ for carbon and 0.37‰ for nitrogen. Precision (based on all four reference materials) was 0.42‰ for carbon and 0.12‰ for nitrogen for protein runs and was 0.04‰ for carbon and 0.11‰ for nitrogen for fecal runs. The average isotopic difference for 24 samples run in duplicate was 0.33 ± 0.35‰ for carbon and 0.30 ± 0.30‰ for nitrogen.

Carbon and oxygen in bone apatite were analyzed at the Light Stable Isotope Mass Spec Lab at the University of Florida on a Finnigan‐MAT 252 isotope ratio mass spectrometer equipped with a Thermo Scientific Kiel III carbonate device. Approximately 0.6 mg of each powdered sample were dissolved in five drops of 100% phosphoric acid at 70°C for 5 min, and the evolved CO_2_ was analyzed. Both carbon and oxygen data are reported relative to VPDB. Precision, based on 19 NBS‐19 replicates, was 0.024‰ for carbon and 0.046‰ for oxygen. The average isotopic difference in δ^13^C and δ^18^O values for seven samples run in duplicate was 0.18 ± 0.11‰ and 0.18 ± 0.16‰, respectively.

Strontium analyses were conducted at the Multicollector ICPMS Laboratory in the Department of Geology at the University of Illinois, Urbana‐Champaign. Three to 5 mg of each sample were dissolved in 0.5 mL of 3 N nitric acid in Teflon tubes. Strontium concentrations were measured for samples collected in 2019 using a 0.5 mL aliquot of each dissolved sample. Samples were analyzed on a Thermo Scientific iCAP Qc quadrupole inductively coupled plasma mass spectrometer (ICP‐MS). Resulting strontium (Sr) concentrations were corrected for initial sample masses and are reported as μg Sr/g sample. Analytical precision (5%) was estimated using 1:100 dilutions of Standard Reference Material (SRM)‐1643f analyzed after every 20 samples. For ^87^Sr/^86^Sr analysis, dissolved samples were filtered through 0.2 mL of Eichrom strontium specific resin in Teflon cation exchange columns, and eluted with 0.05 N nitric acid into 4 mL autosampler vials. Samples were analyzed on a Nu Plasma High Resolution multicollector inductively coupled plasma mass spectrometer (MC‐ICP‐MS). Data were first corrected for mass bias fractionation and then normalized using the international standard SRM 987, and secondary internal laboratory carbonate standards “Coral” and “E&A”. Analytical uncertainty was ≤ ±0.0005.

### 2.3 Data Analysis

We did not attempt to examine data on a day‐by‐day basis but instead compared summary data (combining all days from the study) for each rodent tissue and excreta for each bird. We made this decision because it was apparent that some pellets contained the remains of more than one rodent, and the isotope data clearly indicated the pre‐digested and post‐digested tissues that we sampled during any given 24‐h window were not always from the same consumed rodent. Additionally, there were 2 days when a pellet was not collected from Caspian (see Appendix 2). There were several individual samples that would be considered statistical outliers for some variables for some tissues. However, we chose to include these in our analyses because (1) there was nothing visually unusual about these samples or their respective analytical runs, and (2) excluding these samples did not change the statistical results.

We assessed variance in both isotope values and elemental data (weight %C, weight %N, atomic C:N, and Sr concentration) among groups using Levene tests and compared median isotope values among groups using non‐parametric Wilcoxon tests or Kruskal‐Wallis tests followed by Steel‐Dwass All Pairs Comparisons when appropriate. All statistical analyses were conducted in JMP Pro 16.0 with significance set at α = 0.05. We compared the isotopic and elemental composition of the various excreta textures (tan “mud”, dark brown “crumbles”, “shiny” glassy bits, and the “sulfur‐like” yellow‐gray sample) both before and after acid treatment. We also compared untreated and acidified excreta to the white “urate?” samples. We compared isotopic values among pre‐ and post‐digested rodent tissues and evaluated changes in the isotope values for bone collagen, bone apatite, and fur following digestion for each bird. We also evaluated how comparable data were for the two birds. Finally, we estimated diet‐feces offset for each bird using different rodent tissues (muscle, bone, or muscle + bone). We also compared offsets using either untreated or acidified excreta for carbon and nitrogen, but we were not able to do this for strontium as we did not analyze ^87^Sr/^86^Sr for acidified excreta.

## 3 Results

### 3.1 Similarities and Differences Between Individuals

Overall trends were very similar for Caspian the owl and Rhett the hawk (Table 2, Figure 1). The biggest differences were observed in excreta. Unacidified excreta for Rhett had significantly lower δ^13^C, higher δ^15^N, and considerably more variable weight %N and atomic C:N than for Caspian (Table 2, Figure 1). After acid treatment, significant differences in excreta δ^13^C, δ^15^N and %N remained between individuals, but the difference in atomic C:N disappeared (Table 2, Figure 1). The Sr concentration of unacidified excreta was also apparently more variable for Caspian than Rhett, but the difference was only marginally significant (*p* = 0.058; Table 2). Finally, weight %N for undigested bone was significantly more variable for Rhett than Caspian; fur δ^15^N values were significantly higher, and bioapatite δ^13^C values were also significantly more variable for Rhett both before and after digestion (Table 2). However, these differences likely reflect differences in the individual rats fed to the two birds rather than something related to digestion.

**TABLE 2:** Summary data and Wilcoxon and Levene results for tissue‐specific comparisons between Caspian the eagle owl and Rhett the red‐tailed hawk.

Tissue	Individual	Statistic	*N*	δ^13^C_protein_ (‰)	δ^15^N_protein_ (‰)	Weight %C	Weight %N	Atomic C:N	δ^13^C_apatite_ (‰)	δ^18^O_apatite_ (‰)	^87^Sr/^86^Sr	Sr Conc (μg Sr/g sample)
Predigestion												
Bone	Caspian	Mean	11	−19.3	5.7	47.9	14.4	3.9	−12.3	−4.4	0.70941	125.0
		Median		−19.4	5.7	49	14.4	3.8	−12.1	−4.2	0.70964	120.1
		SD		0.5	0.3	2.6	0.8	0.3	0.6	1.1	0.00045	40.0
	Rhett	Mean	11	−19.5	5.5	45.3	13.0	4.1	−11.4	−4.0	0.70969	106.6
		Median		−19.5	5.4	46.6	14	4.1	−11.8	−3.6	0.70972	101.1
		SD		0.8	0.5	5.0	1.9	0.4	1.6	0.9	0.00035	11.2
				χ^2^ = 0.21, *p* = 0.64	χ^2^ = 0.74, *p* = 0.39	χ^2^ = 1.40, *p* = 0.24	χ^2^ = 3.03, *p* = 0.082	χ^2^ = 2.11, *p* = 0.15	χ^2^ = 1.8, *p* = 0.18	χ^2^ = 1.40, *p* = 0.24	χ^2^ = 2.09, *p* = 0.15	χ^2^ = 0.71, *p* = 0.40
				Levene *p* = 0.22	Levene *p* = 0.29	Levene *p* = 0.056	**Levene *p* = 0.0096**	Levene *p* = 0.61	**Levene *p* = 0.020**	Levene *p* = 0.78	Levene *p* = 0.31	Levene *p* = 0.095
Fur	Caspian	Mean	11	−19.2	6.1	48.6	14.7	3.9				
		Median		−19.2	6.1	48.5	14.5	3.9				
		SD		0.1	0.2	1.3	0.6	0.2				
	Rhett	Mean	11	−18.9	5.8	47.9	14.7	3.8				
		Median		−19.1	5.8	48.2	14.8	3.8				
		SD		0.5	0.3	1.8	0.6	0.1				
				χ^2^ = 0.64, *p* = 0.42	**χ** ^**2**^ **= 7.17, *p* = 0.0074**	χ^2^ = 0.68 *p* = 0.41	χ^2^ = 0.070, *p* = 0.79	χ^2^ = 1.21 *p* = 0.27				
				**Levene *p* = 0.019**	Levene *p* = 0.93	Levene *p* = 0.91	Levene *p* = 0.84	Levene *p* = 0.20				
Muscle	Caspian	Mean	11	−20.6	6.3	51.8	14.3	4.2				
		Median		−20.8	6.5	51.4	14.2	4.2				
		SD		0.5	0.4	6.4	1.7	0.2				
	Rhett	Mean	11	−20.4	6.1	51.0	14.0	4.3				
		Median		−20.6	6	51.4	14	4.3				
		SD		0.9	0.3	1.8	0.6	0.3				

				χ^2^ = 0.16, *p* = 0.69	χ^2^ = 2.95, *p* = 0.086	χ^2^ = 0.0011, *p* = 0.97	χ^2^ = 0.11, *p* = 0.74	χ^2^ = 0.58 *p* = 0.45				
Levene *p* = 0.15				Levene *p* = 0.33	Levene *p* = 0.18	Levene *p* = 0.28	Levene *p* = 0.55				
Post digestion												
Bone	Caspian	Mean	9	−18.2	5.6	45.0	15.5	3.4	−13.2	−4.0	0.70815	59.6
		Median		−18.2	5.7	45.8	15.5	3.4	−13.25	−4.1	0.70811	59.0
		SD		0.3	0.4	3.7	0.6	0.2	0.6	0.6	0.00011	6.2
	Rhett	Mean	5	−18.0	5.2	44.5	14.7	3.5	−11.9	−4.7	0.70836	61.3
		Median		−17.9	5.2	46.9	15.0	3.6	−12.5	−4.6	0.70821	62.4
		SD		0.3	0.3	5.3	1.0	0.3	1.7	1.6	0.00034	6.4
				χ^2^ = 0.90, *p* = 0.34	χ^2^ = 2.83, *p* = 0.093	χ^2^ = 0.040, *p* = 0.84	χ^2^ = 2.2, *p* = 0.14	χ^2^ = 2.20, *p* = 0.14	χ^2^ = 2.66, *p* = 0.10	**χ** ^**2**^ **= 1.63, *p* = 0.02**	χ^2^ = 2.88, *p* = 0.090	χ^2^ = 0.38, *p* = 0.54
				Levene *p* = 0.89	Levene *p* = 0.65	Levene *p* = 0.053	Levene *p* = 0.22	Levene *p* = 0.71	**Levene *p* = 0.043**	Levene *p* = 0.11	**Levene *p* = 0.016**	Levene *p* = 0.96
Fur	Caspian	Mean	9	−19.2	6.0	44.9	13.5	3.9				
		Median		−19.2	6.0	45.3	13.5	3.8				
		SD		0.2	0.3	2.4	0.7	0.2				
	Rhett	Mean	11	−19.2	5.6	46.9	14.0	3.9				
		Median		−19.0	5.5	47.7	14.1	4.0				
		SD		0.3	0.4	3.3	0.8	0.2				
				χ^2^ = 0.24, *p* = 0.88	**χ** ^**2**^ **= 5.41, *p* = 0.02**	χ^2^ = 2.43, *p* = 0.12	χ^2^ = 2.68, *p* = 0.10	χ^2^ = 0.55 *p* = 0.48				
				**Levene *p* = 0.0082**	Levene *p* = 0.50	Levene *p* = 0.24	Levene *p* = 0.81	Levene *p* = 0.61				
Unacidified excreta	Caspian	Mean	13	−21.5	6.2	41.6	6.2	7.2			0.708534	31.6
		Median		−21.3	6.6	45.8	5.0	7.2			0.70850	32.2
		SD		1.1	1.2	14.2	2.3	2.2			0.00026	25.7
Unacidified excreta
	Rhett	Mean	11	−20.6	4.7	38.7	2.9	18.2			0.70871	57.8
		Median		−20.5	4.4	46.0	2.4	17.1			0.70876	59.0
		SD		0.4	1.0	14.6	1.4	9.4			0.00025	14.3
				χ ^2^ = 3.72, *p* = 0.058	**χ** ^**2**^ **= 7.02, *p* = 0.0081**	χ^2^ = 0.22, *p* = 0.64	**χ** ^**2**^ **= 10.56, *p* = 0.0012**	χ^2^ = 3.23, *p* = 0.072			χ^2^ = 2.38, *p* = 0.12	χ ^2^ = 3.57, *p* = 0.058
**Levene *p* = 0.038**				Levene *p* = 0.28	Levene *p* = 0.12	**Levene *p* = 0.012**	**Levene *p* < 0.0001**			Levene *p* = 0.69	**Levene *p* = 0.022**
Acidified excreta	Caspian	Mean	12	−22.3	5.4	55.9	11.3	6.8				
		Median		−22.5	5.4	57.9	8.9	7.7				
		SD		1.1	0.8	6.5	6.6	2.2				
	Rhett	Mean	10	−21.2	4.6	53	7.5	8.3				
		Median		−21.2	4.6	52.3	7.7	8.0				
		SD		0.5	0.4	3.8	0.8	1.1				
				**χ** ^**2**^ **= 8.25, *p* = 0.0041**	**χ** ^**2**^ **= 4.74, *p* = 0.030**	χ^2^ = 2.72, *p* = 0.099	**χ** ^**2**^ **= 10.68, *p* = 0.0011**	χ^2^ = 2.21, *p* = 0.14				
				Levene *p* = 0.21	**Levene *p* = 0.012**	Levene *p* = 0.30	Levene *p* = 0.063	Levene *p* = 0.097				

*Note:* Significant results are presented in bold and marginally significant results are underlined.

### 3.2 Differences Among Tissues for Consumed Prey

**FIGURE 2:**
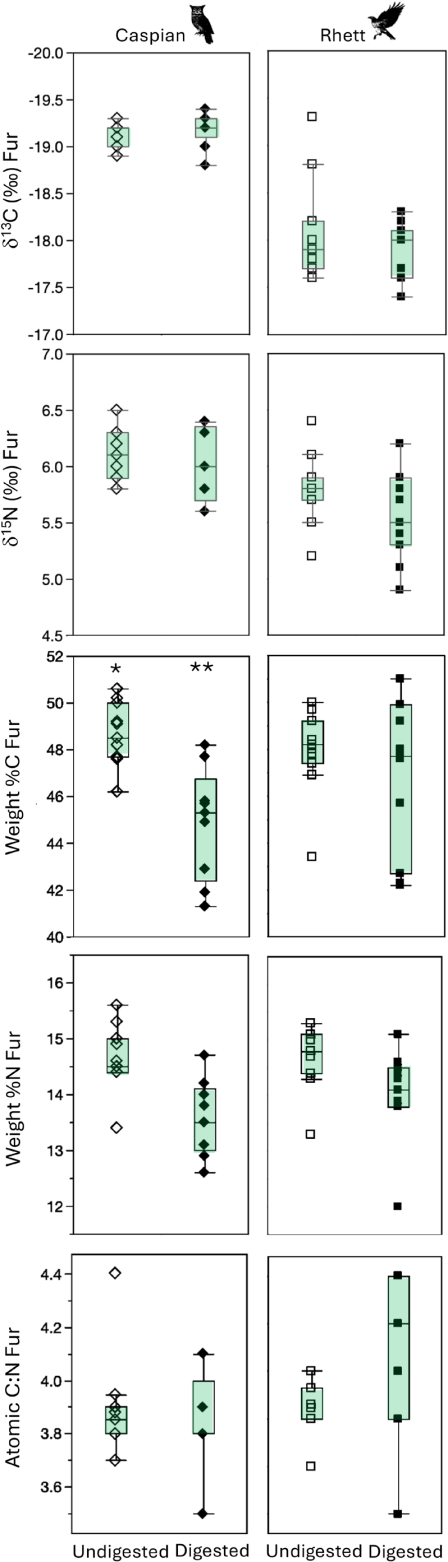
Box plots comparing the δ^13^C, δ^15^N, and elemental composition of undigested rodent fur (fed to Caspian the eagle owl and Rhett the red‐tailed hawk), and digested rodent fur dissected from pellets regurgitated by the two birds. Asterisks indicate significant differences between undigested and digested tissues based on Steel‐Dwass all pairs post hoc comparisons among tissues for each bird (Table 3).

**FIGURE 3:**
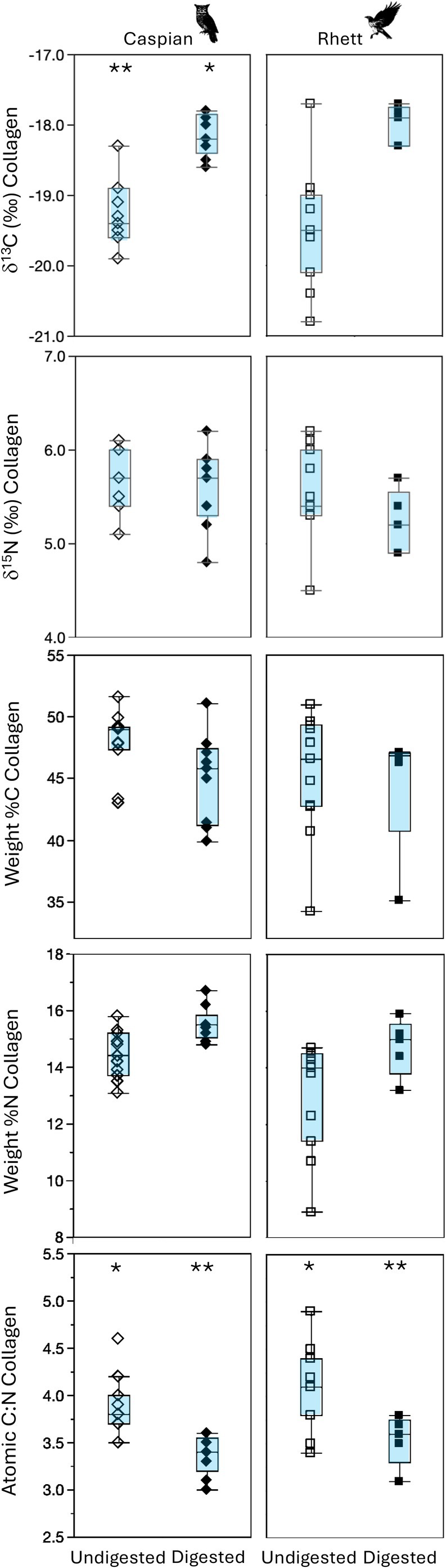
Box plots comparing the δ^13^C, δ^15^N, and elemental composition of undigested and digested rodent bone collagen (sampled prior to feeding to Caspian the eagle owl and Rhett the red‐tailed hawk and then dissected from pellets regurgitated by the two birds). Asterisks indicate significant differences between undigested and digested tissues based on Steel‐Dwass all pairs post hoc comparisons among tissues for each bird (Table 3).

There were significant differences among rodent tissues for most isotopes both before and after digestion. Looking first at proteinaceous tissues, there were significant differences in median δ^13^C values among tissues for both Caspian the owl and Rhett the hawk (Table 3; Figure 1). Post hoc tests indicate that digested bone collagen had the highest δ^13^C values, undigested bone collagen and both undigested and digested fur had intermediate values, and muscle had the lowest tissue δ^13^C values for both birds. Rodent fur δ^13^C values did not change with digestion, but bone collagen increased by ca. 1.5‰ following digestion for both birds (Figures 2 and 3).

**TABLE 3:** Summary statistics for rodent proteinaceous tissues and excreta from Caspian the eagle owl and Rhett the red‐tailed hawk.

Individual	Tissue	*N*	δ^13^C (‰)			δ^15^N (‰)			Weight %C			Weight %N			Atomic C:N		
			Mean	SD	Median	Mean	SD	Median	Mean	SD	Median	Mean	SD	Median	Mean	SD	Median
Caspian																	
Predigestion	Bone	11	−19.3	0.5	−19.4^b^	5.7	0.3	5.7^b^	47.9	2.6	49^bc^	14.4	0.8	14.4^ab^	3.9	0.3	3.8^b^
	Fur	11	−19.1	0.1	−19.2^b^	6.1	0.2	6.1^ab^	48.6	1.3	48.5^ac^	14.7	0.6	14.5^ab^	3.9	0.2	3.9^b^
	Muscle	11	−20.6	0.5	−20.8^c^	6.3	0.4	6.5^a^	51.8	6.4	51.4^ac^	14.3	1.6	14.2^abc^	4.2	0.2	4.2^b^
Postdigestion	Bone	9	−18.2	0.3	−18.2^a^	5.6	0.4	5.7^ab^	45.0	3.7	45.8^bc^	15.5	0.6	15.5^b^	3.4	0.2	3.4^c^
	Fur	9	−19.2	0.2	−19.2^b^	6	0.3	6.0^ab^	44.9	2.4	45.3^b^	13.5	0.7	13.5^abc^	3.9	0.2	3.8^b^
	Unacidified excreta	13	−21.7	0.9	−21.5^d^	6.4	1	6.6^ab^	41.6	14.2	45.8^bc^	6.2	2.3	5^d^	8.5	3.9	7.1^a^
	Acidified excreta	12	−22.3	1.1	−22.5^d^	5.4	0.8	5.4^ab^	55.9	6.5	57.9^a^	11.3	6.6	8.9^cd^	6.8	2.2	7.7^a^
					**χ** ^**2**^ **= 64.48, df = 6, *p* < 0.0001**			**χ** ^**2**^ **= 25.99, df = 6, *p* = 0.0002**			**χ** ^**2**^ **= 31.67, df = 6, *p* < 0.0001**			**χ** ^**2**^ **= 51.72, df = 6, *p* < 0.0001**			**χ** ^**2**^ **= 53.95, df = 6, *p* < 0.0001**
					**Levene *p* = 0.0013**			**Levene *p* = 0.0015**			**Levene *p* < 0.0001**			**Levene *p* = 0.002**			**Levene *p* < 0.0001**
Rhett																	
Predigestion	Bone	11	−19.4	0.8	−19.5^ab^	5.5	0.5	5.4^a^	45.3	5	46.6^b^	13.0	1.9	14.0^a^	4.1	0.4	4.1^bc^
	Fur	11	−18.9	0.5	−19.1^ab^	5.8	0.3	5.8^a^	47.9	1.8	48.2^b^	14.0	0.6	14.0^a^	3.8	0.07	3.8^c^
	Muscle	11	−20.4	0.9	−20.6^bc^	6.1	0.3	6^a^,*	51.0	1.8	51.4^ac^	14.0	0.6	14.0^a^	4.3	0.3	4.3^b^
Postdigestion	Bone	5	−18.0	0.3	−17.9^a^	5.2	0.3	5.2^a^,*	44.5	5.3	46.9^bc^	14.7	1.0	15.0^a^	3.5	0.3	3.6^bc^
	Fur	11	−19.1	0.3	−19.0^b^	5.6	0.4	5.5^a^	46.9	3.3	47.7^b^	14.0	0.8	14.1^a^	3.9	0.2	4.0^c^
	Unacidified excreta	11	−20.7	0.4	−20.5^c^	4.6	0.7	4.4^b^	38.7	14.6	46.0^b^	2.9	1.4	2.4^c^	18.2	9.4	22.1^a^
	Acidified excreta	10	−21.2	0.5	−21.2^c^	4.6	0.4	4.6^b^	53.0	3.9	52.3^a^	7.5	0.8	7.7^b^	8.3	1.1	8.0^a^
					**χ** ^**2**^ **= 48.80, df = 6, *p* < 0.0001**			**χ** ^**2**^ **= 40.93, df = 6, *p* < 0.0001**			**χ** ^**2**^ **= 35.93, df = 6, *p* < 0.0001**			**χ** ^**2**^ **= 50.92, df = 6, *p* = 0.0007**			**χ** ^**2**^ **= 52.45, df = 6, *p* < 0.0001**
					Levene *p* = 0.23			Levene *p* = 0.20			**Levene *p* < 0.0001**			**Levene *p* < 0.0001**			**Levene *p* < 0.0001**

*Note:* Significant tests are presented in bold. Tissues that share a superscript letter within each comparison are stastistically indisinguishable using Steel–Dwass all pairs tests.

* δ^15^N for muscle and digested bone were nearly significanly different for Rhett (= 0.058).

There were also significant differences in δ^15^N values among rodent tissues for both Rhett and Caspian (Table 3; Figure 1). Post hoc tests indicate a significant difference between muscle and undigested bone for Caspian and a marginally significant (*p* = 0.058) difference in δ^15^N values between muscle and digested bone for Rhett. Muscle had relatively elevated δ^15^N values, undigested and digested fur had intermediate values, and undigested and digested collagen had lower δ^15^N values than other rodent tissues for both birds. Nitrogen isotope values in both fur and collagen were unaffected by digestion (Figures 2 and 3).

Finally, there were small but significant differences in weight %C and atomic C:N, as well as apparent differences in %N among rodent tissues for both birds (Table 3; Figures 1, 2, 3). For Caspian, post hoc tests indicate that digested fur had distinct %C from either undigested fur or muscle, and digested bone had significantly smaller atomic C:N than the other proteinaceous tissues, including undigested bone (Table 3; Figure 1). For Rhett, the only significant pairwise comparisons were muscle had distinct weight %C from all other tissues except digested bone, and significantly larger atomic C:N than both digested and undigested fur (although digested bone had apparently smaller C:N than undigested bone; 3.6 vs. 4.1; Figure 1; Table 3). There was a significant decrease in weight %C with digestion for fur for Caspian, and an apparent but insignificant decrease in weight %N for fur as well as a decrease in %C and an increase in %N for bone collagen for both birds (Figures 2 and 3). Atomic C:N was significantly larger for undigested than digested collagen for both birds (Figure 3).

**FIGURE 4:**
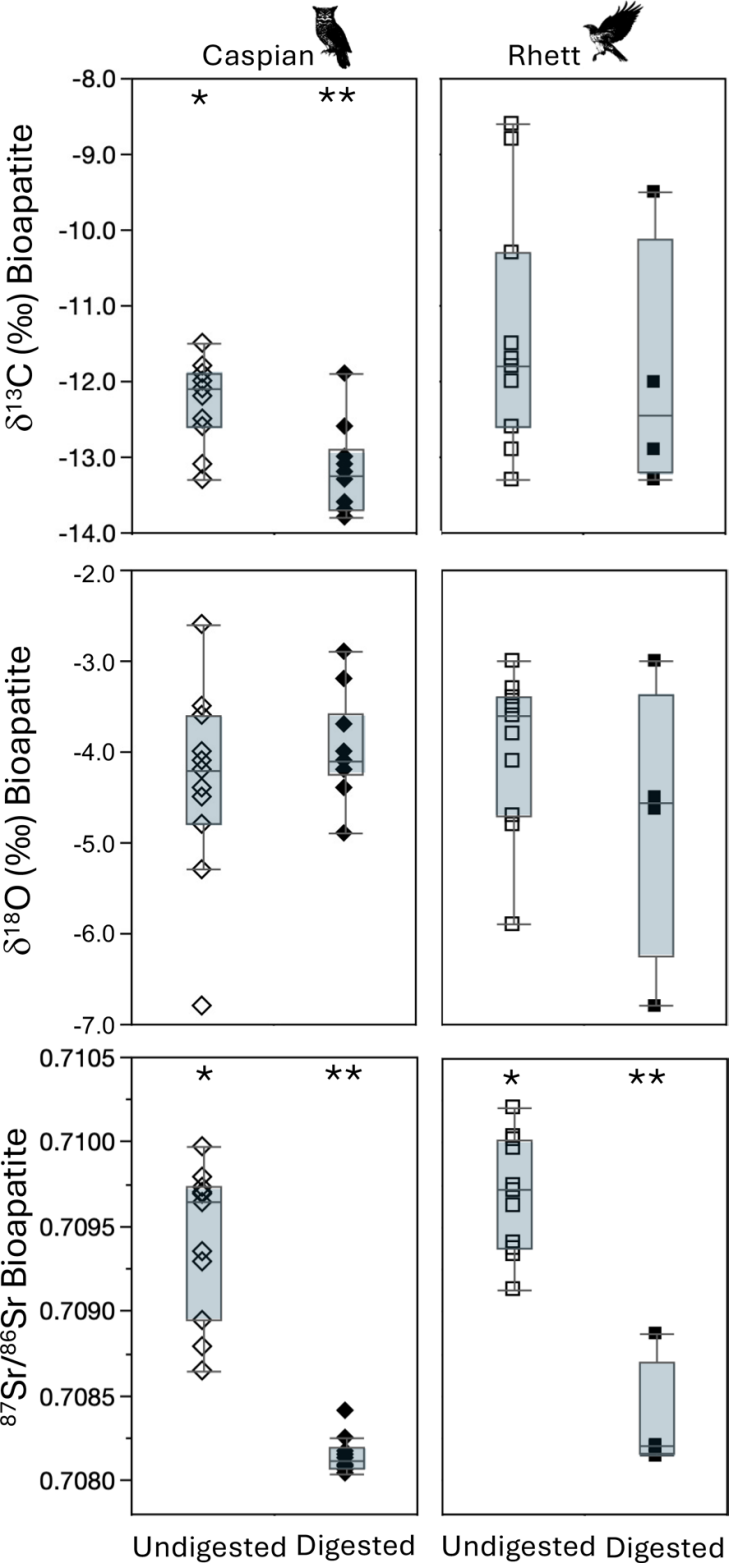
Box plots comparing δ^13^C, δ^18^O, and ^87^Sr/^86^Sr for isotopic and elemental composition of undigested rodent tissues (sampled prior to feeding to Caspian the eagle owl and Rhett the red‐tailed hawk and then dissected from pellets regurgitated by the two birds). Asterisks indicate significant differences between undigested and digested tissues based on Steel‐Dwass all pairs post hoc comparisons among tissues for each bird (Table 3).

Moving on to rodent bioapatite, we found significantly lower δ^13^C values in digested than undigested bioapatite for Caspian (Figure 4; Table 4). The overall range in δ^13^C for both digested and undigested bioapatite was much larger for Rhett, and the two groups were statistically indistinguishable. Oxygen isotope values in rodent bioapatite were statistically indistinguishable before and after digestion by both birds (Figure 4; Table 4). Finally, median ^87^Sr/^86^Sr for undigested rodent bioapatite was significantly higher than that for digested bioapatite for both birds, and undigested bioapatite also had significantly more variable ^87^Sr/^86^Sr than digested bioapatite for Caspian (Figure 4; Table 4). Strontium concentration was also significantly larger for undigested than for digested bioapatite for both birds (Table 4).

**TABLE 4:** Summary statistics for rodent bioapatite δ^13^C and δ^18^O, and ^87^Sr/^86^Sr and strontium data for excreta from Caspian the eagle owl and Rhett the red‐tailed hawk.

Individual	Tissue full	δ^13^C (‰)				δ^18^O (‰)				^87^Sr/^86^Sr				Sr Concentration (μg Sr/g sample)			
		*N*	Mean	SD	Median	*N*	Mean	SD	Median	*N*	Mean	SD	Median	*N*	Mean	SD	Median
Caspian	Undigested bone	11	−12.3	0.6	−12.1^a^	11	−4.3	1.1	−4.2^a^	11	0.70941	0.00045	0.70964^a^	8	125.02	40.04	120.07^a^
	Digested bone	10	−13.2	0.6	−13.3^b^	10	−4	0.6	−4.1^a^	10	0.70815	0.00011	0.70811^b^	8	59.64	6.16	59.03^b^
	Unacidified excreta									11	0.70854	0.00026	0.70850^b^	8	31.59	25.72	32.20^b^
					**χ** ^**2**^ **= 6.67, df = 1, *p* = 0.0098**				χ^2^ = 0.64, df = 1, *p* = 0.43				**χ** ^**2**^ **= 24.09; df = 2; *p* < 0.0001**				**χ** ^**2**^ **= 17.79, df = 2, *p* = 0.0001**
					Levene *p* = 0.87				Levene *p* = 0.30				**Levene *p* = 0.0011**				Levene *p* = 0.037
Rhett	Undigested bone	11	−11.4	1.6	−11.8^a^	11	−4.0	0.9	−3.6^a^	11	0.70969	0.00035	0.70972^a^	8	106.63	11.16	101.12^a^
	Digested bone	4	−11.9	1.7	−12.5^a^	4	−4.7	1.6	−4.6^b^	4	0.70836	0.00034	0.70821^b^	3	61.32	6.37	62.38^b^
	Unacidified excreta									11	0.70871	0.00025	0.70876^b^	8	57.83	14.3	59.01^b^
					χ^2^ = 0.73, df = 1, *p* = 0.39				χ^2^ = 0.15, df = 1, *p* = 0.70				**χ** ^**2**^ **= 19.65; df = 2; *p* < 0.0001**				**χ** ^**2**^ **= 13.26, df = 2, *p* = 0.0013**
					Levene *p* = 0.48				Levene *p* = 0.089				Levene *p* = 0.53				Levene *p* = 0.19

*Note:* Significant tests are presented in bold. Tissues that share a superscript letter within each comparison are stastistically indisinguishable using Steel–Dwass all pairs tests.

### 3.3 Trends for Excreta

**FIGURE 5:**
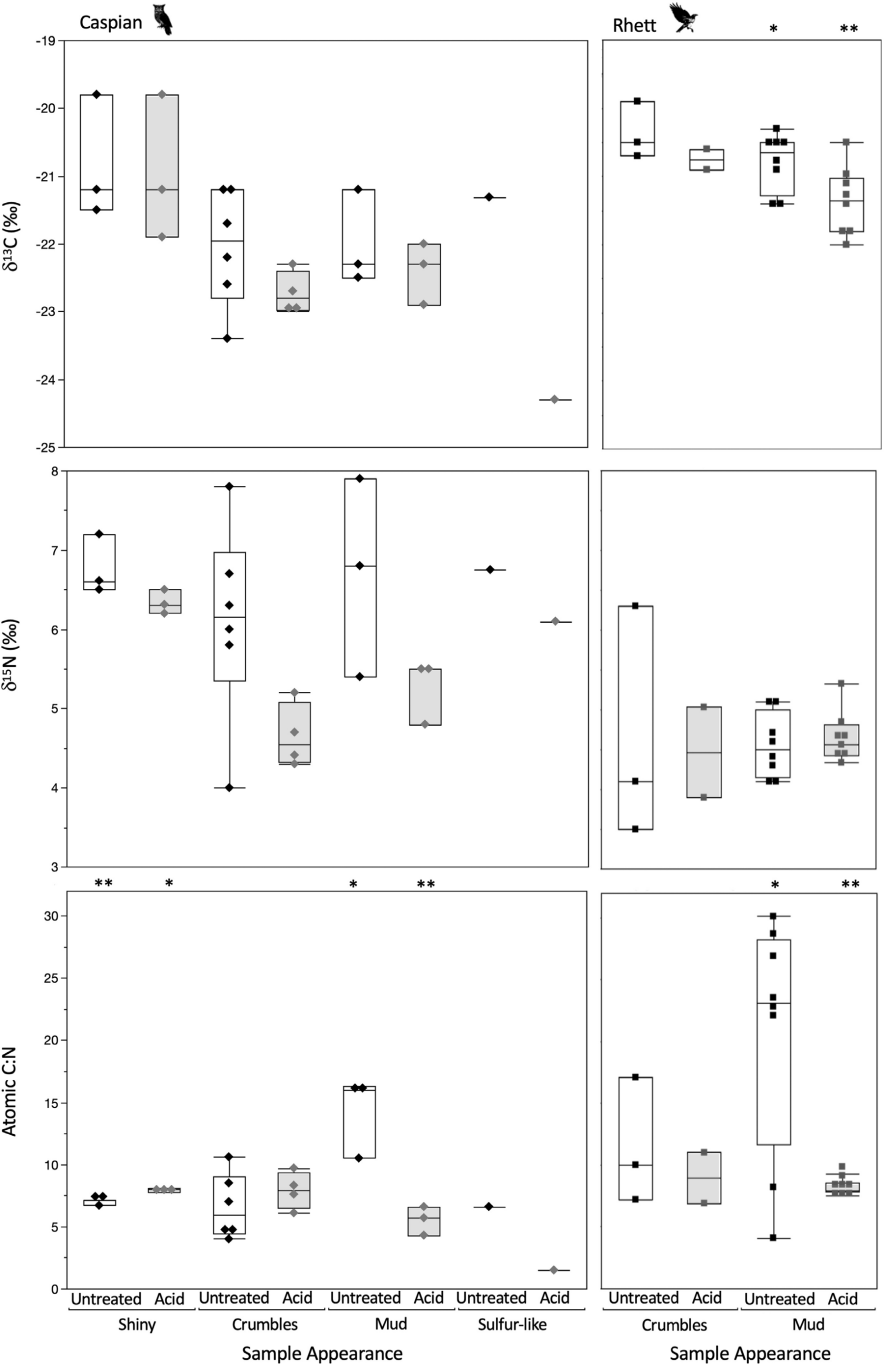
Box plots comparing δ^13^C and δ^15^N values for untreated and acidified raptor excreta with differing appearances for Caspian the eagle owl and Rhett the red‐tailed hawk. Asterisks denote significant differences between unacidified and acidified samples. Summary data and statistical comparisons of excreta textures for each individual are provided in Appendix 3.

Looking first at unacidified samples, we found no differences in median δ^13^C, δ^15^N, weight %C, or atomic C:N values among the various excreta textures for either Caspian or Rhett (Figure 5; Appendix 3). We found significant differences in %N among excreta textures for Caspian. However, post hoc tests failed to find any pairwise differences; “shiny” samples had apparently but insignificantly larger %N, and “mud” samples had apparently smaller %N than other textures (Appendix 3). “Mud” samples had apparently larger atomic C:N than other textures for both individuals (Figure 5; Appendix 3). There were significant differences in variance among excreta textures for weight %C for Caspian, and atomic C:N as well as apparent but insignificant differences in weight %N for both birds (Figure 5; Appendix 3). “Shiny” samples tended to have less variable elemental data than other textures, while “mud” and “crumbles” alternately had more variable values. Finally, there were no significant differences in ^87^Sr/^86^Sr or Sr concentration among unacidified excreta textures for either bird (Appendix 3), but there were pronounced differences in Sr concentrations for Caspian. “Shiny” excreta had ca. 10× lower Sr concentrations than the other three texture types (ca. 5 vs. 40–60 μg Sr/g sample; Appendix 3).

Acidifying excreta had a notable influence on weight %C, %N, atomic C:N, δ^13^C, and δ^15^N values for some, but not all of the excreta textures (Figure 5; Appendix 3). There were many visibly clear (in some cases considerable) changes in δ^13^C and δ^15^N values for most of the textures, but only three of these were significant. There was a significant decline in δ^13^C values for “mud” excreta for Rhett. “Mud” excreta also had smaller and significantly less variable atomic C:N after acidification for both birds. Finally, there was a small but significant increase in atomic C:N for “shiny” samples for Caspian (Figure 5). There were significant differences in both δ^13^C and δ^15^N values among acidified excreta textures for Caspian (Figure 5; Appendix 3). Post hoc tests failed to detect any pairwise differences for either isotope, but the single “sulfur‐like” sample had apparently lower δ^13^C, and the “shiny” excreta had apparently higher δ^13^C values than the other sample types. There were no isotopic differences among excreta textures for Rhett. There were also no differences in median weight %C, %N, or atomic C:N among acidified excreta textures for either bird. However, there were differences in variance for weight %N for both birds, and atomic C:N for Caspian (Figure 5; Appendix 3). Similar to the patterns observed for unacidified excreta, “shiny” samples tended to have less variable elemental data than other textures.

**FIGURE 6:**
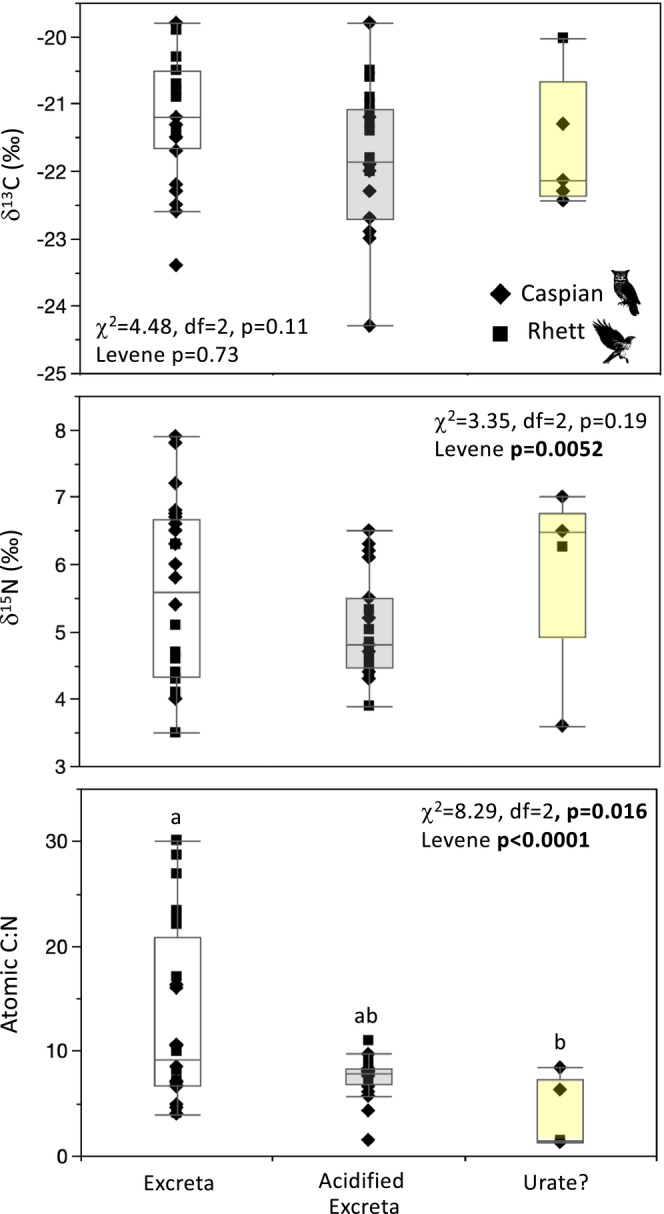
Box plots comparing δ^13^C, δ^15^N and atomic C:N for unacidified excreta, acidified excreta, and white flecks (possibly urate?) isolated from excreta samples for Caspian the eagle owl and Rhett the red‐tailed hawk. Significant statistical results are presented in bold.

The five “urate?” samples tended to have elevated weight %N (Appendix 3) and all had atomic C:N between 1.3 and 8.4 (significantly smaller than unacidified feces but indistinguishable from acidified feces; Figure 6; Appendix 3). These samples had statistically indistinguishable δ^13^C and δ^15^N values from both unacidified and acidified excreta. However, there was a rather large spread in δ^15^N values (four samples had δ^15^N values between ca. 6 and 7‰ while one had a value closer to 3.5‰; Figure 6; Appendix 3). Given how few of the “urate?” samples we had available, we excluded them from further statistical analyses and figures. However, we do include these samples in the Discussion.

### 3.4 Estimating Diet‐Excreta Offset (Δ_diet‐excreta_)

As discussed above, we were unable to confidently isolate feces from other waste products and therefore use “excreta” to acknowledge the possible contribution of urine or urates to our offset estimates. Unacidified excreta had significantly lower δ^13^C values than any of the proteinaceous rodent tissues for Caspian, and significantly lower δ^13^C values than any of the rodent tissues except muscle for Rhett (Table 3). However, trends for nitrogen diverged for the two birds. For Caspian, unacidified excreta had relatively elevated δ^15^N values and was statistically indistinguishable from all rodent tissues. For Rhett, unacidified excreta δ^15^N values were significantly lower than all rodent tissues (Table 3). Acidified excreta had statistically indistinguishable δ^13^C and δ^15^N values from unacidified excreta, although they did have slightly different average values (ca. ≤ 1‰; Table 3). This was especially evident for nitrogen isotope values for Caspian. Unacidified excreta ^87^Sr/^86^Sr and Sr concentrations were both significantly lower than undigested rodent bioapatite, but statistically indistinguishable from digested bioapatite for both Caspian and Rhett (Table 4).

**FIGURE 7:**
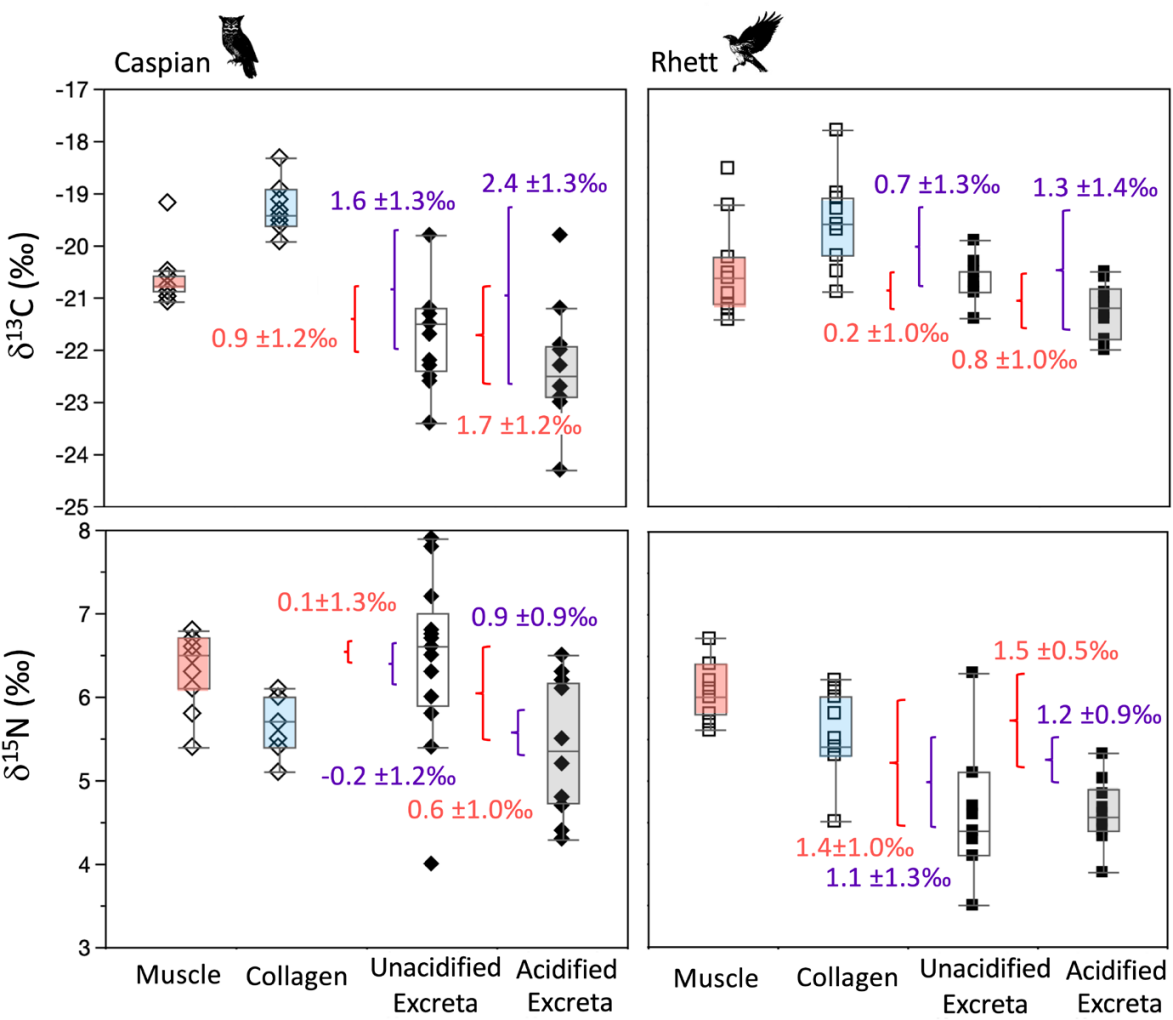
Box plots and summary estimates (mean ± 1*σ*) of Δ^13^C_diet‐excreta_ and Δ^15^N_diet‐excreta_ for Caspian the eagle owl and Rhett the red‐tailed hawk based on just consumed rat muscle (in red), or rat muscle + collagen (in purple).

Because raptors can, and do, digest bone, we provide multiple different estimates of Δ_diet‐excreta_ using the isotopic offset between rodent muscle or bone collagen + muscle, and both unacidified and acidified excreta. While excreta δ^13^C values for both birds tended to be lower than those for proteinaceous rodent tissues, Δ^13^C_diet‐excreta_ estimates were rather different for the two birds (summarized in Figure 7). First, using unacidified feces and assuming muscle was the primary contributor to dietary C, average Δ^13^C_diet‐excreta_ (± 1 standard deviation) was 0.9 ± 1.2‰ and 0.2 ± 1.0‰ for Rhett and Caspian, respectively (Figure 7). Assuming dietary C was drawn from both muscle and collagen, these values increased slightly (1.5 ± 1.3‰ for Caspian and 0.7 ± 1.3‰ for Rhett). Using acidified feces increases offset estimates as well. Assuming muscle was the primary contributor to dietary C, then average Δ^13^C_diet‐feces_ was 1.7 ± 1.2‰ and 0.8 ± 1.0‰ for Rhett and Caspian, respectively. Lastly, assuming both muscle and collagen contributed to diet, these values increased to 2.4 ± 1.3‰ for Caspian and 1.3 ± 1.4‰ for Rhett (Figure 7).

Diet‐excreta offset for nitrogen was markedly different between the two individuals. Working with unacidified feces and assuming dietary nitrogen was derived from muscle, Δ^15^N_diet‐excreta_ was 0.1 ± 1.3‰ for Caspian and 1.4 ± 1.0‰ for Rhett, respectively (Figure 7). Assuming both muscle and collagen contributed, diet‐feces offset was −0.2 ± 1.2‰ for Caspian and 1.1 ± 1.3‰ for Rhett. Switching to acidified excreta, estimated offset values increased for Caspian but were not noticeably different for Rhett. Assuming dietary N came exclusively from muscle, the estimated offset was 0.9 ± 0.9‰ for Caspian and 1.5 ± 0.5‰ for Rhett. And finally, assuming both muscle and bone collagen contributed, the estimated offset was 0.6 ± 1.0‰ for Caspian and 1.2 ± 0.9‰ for Rhett (Figure 7).

**FIGURE 8:**
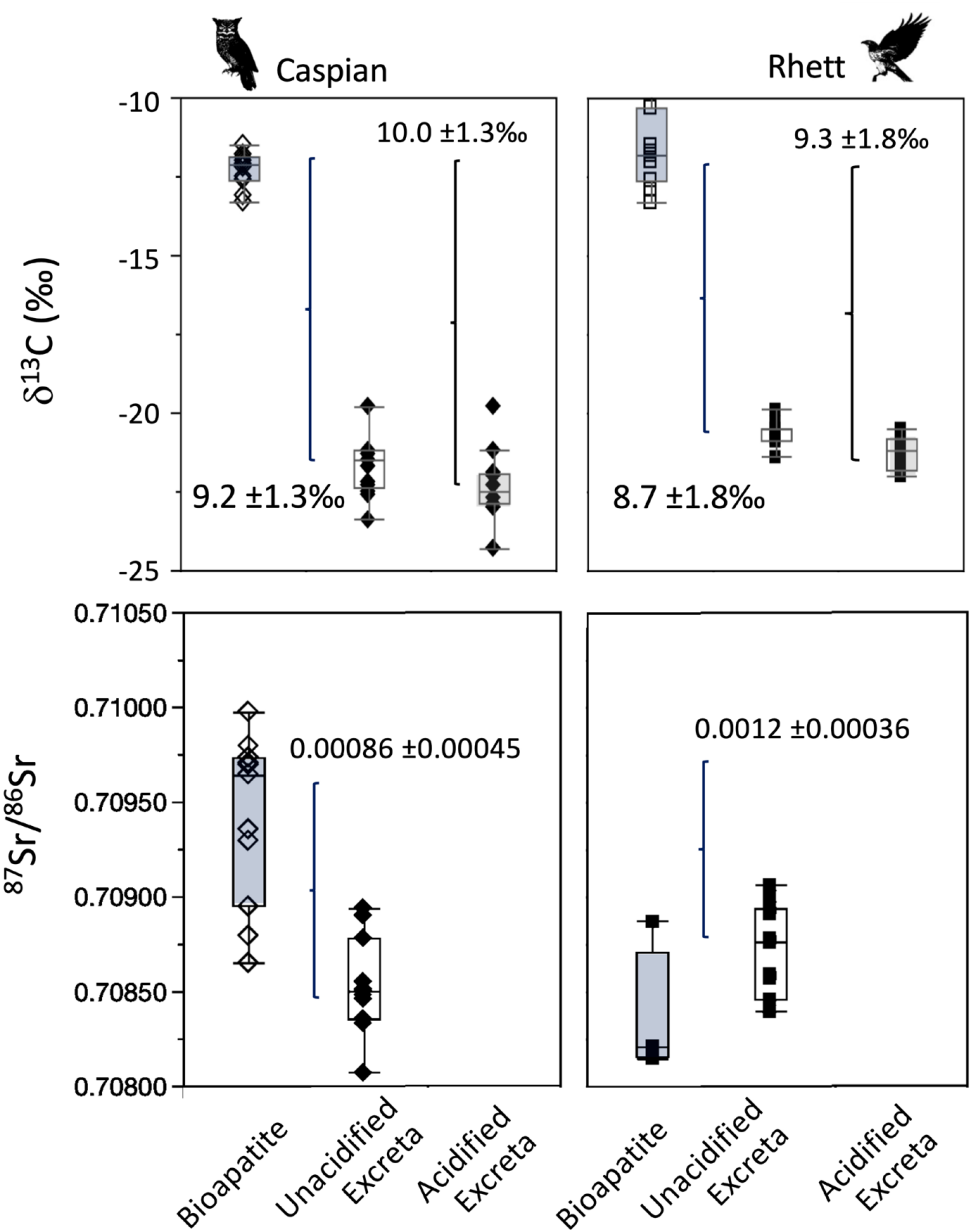
Box plots and summary estimates (mean ± 1*σ*) of Δ^13^C_diet‐excreta_ and Δ^87^Sr/^86^Sr_diet‐excreta_ for Caspian the eagle owl and Rhett the red‐tailed hawk based on bone bioapatite of consumed rats. We were not able to analyze strontium isotopes for acidified excreta.

Average Δ^13^C_diet‐excreta_ based on bioapatite shifted from 9.2 ± 1.3‰ to 10.0 ± 1.3‰ for Caspian and 8.7 ± 1.8‰ to 9.3 ± 1.8‰ for Rhett when using unacidified and acidified excreta, respectively (Figure 8). Lastly, Δ^87^Sr/^86^Sr_diet‐excreta_ based on unacidified excreta was positive for both birds and slightly smaller for Caspian than Rhett (0.0086 ± 0.00045 and 0.0012 ± 0.00036, respectively; Figure 8).

## 4 Discussion

### 4.1 Diet‐Excreta Offset for Raptors

Using proteinaceous rodent tissues as a proxy for raptor diet, Δ^13^C_diet‐excreta_ ranged from 0.9 ± 1.2‰ to 2.4 ± 1.3‰ for Caspian and 0.2 ± 1.0‰ to 1.3 ± 1.4‰ for Rhett depending on if we used just muscle or both muscle and collagen, and if excreta had been acidified (Figure 7). For both birds, Δ^13^C_diet‐excreta_ was larger when both collagen and muscle were considered to contribute to diet, and offset was larger in acidified than unacidified excreta. Average Δ^15^N_diet‐feces_ ranged from −0.2 ± 1.2‰ to 0.9 ± 0.9‰ for Caspian and 1.1 ± 1.3‰ to 1.5 ± 0.5‰ for Rhett (Figure 7). For both birds, Δ^15^N_diet‐feces_ was consistently smaller (by 0.3‰) when diet included both muscle and bone. Acidifying excreta increased Δ^15^N_diet‐excreta_ by ca. 0.8‰ for Caspian but had a negligible impact on Δ^15^N_diet‐excreta_ for Rhett.

**FIGURE 9:**
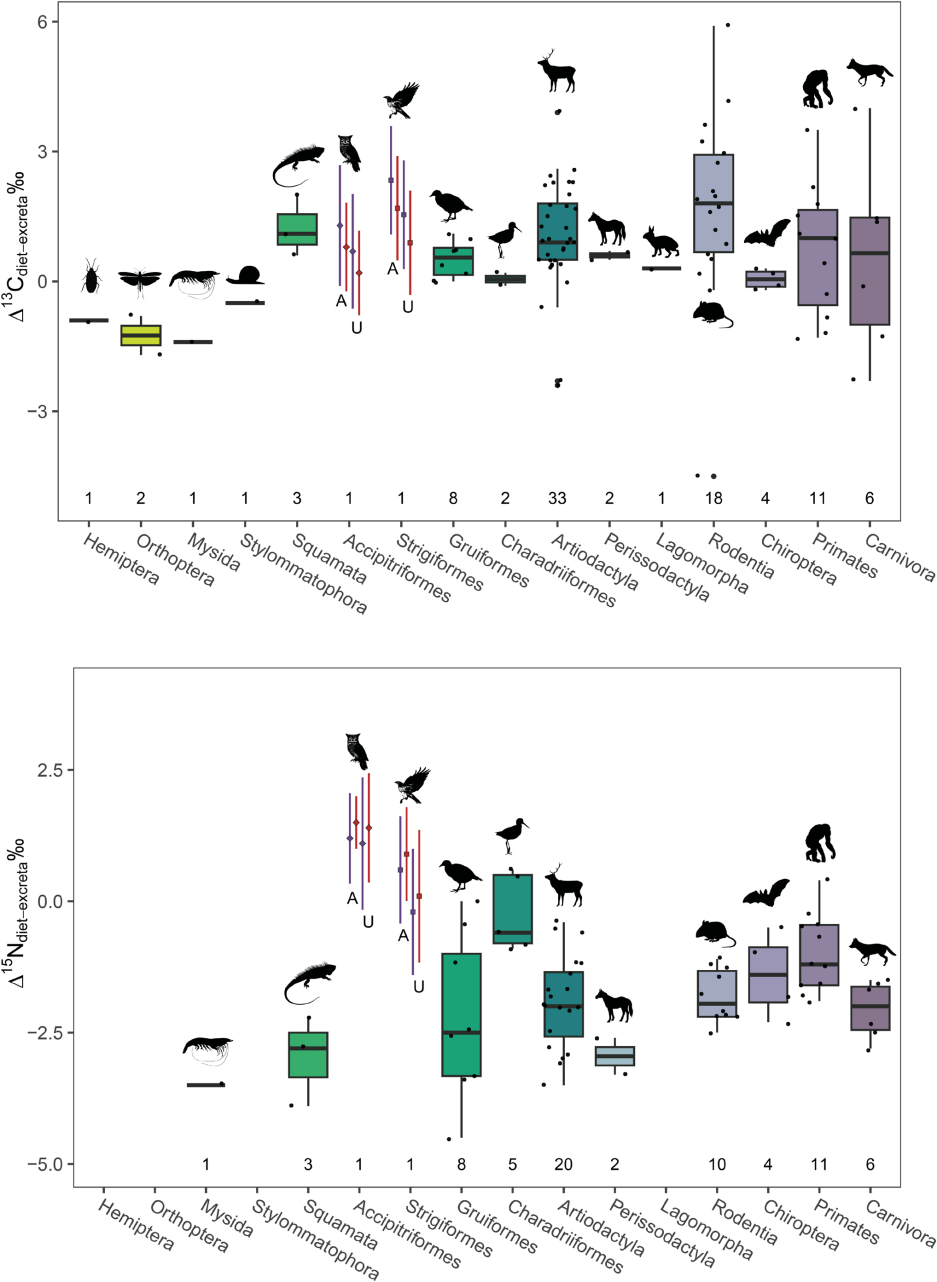
Comparison of mean and standard deviation Δ^13^C_diet‐excreta_ and Δ^15^N_diet‐excreta_ for Rhett and Caspian with previously published offsets for a broad array of organisms (adapted from Reid et al. 2023). Numbers indicate number of individuals included for each taxon. Following Figure 7, offsets calculated using just rat muscle are in red and those calculated using rat muscle + bone collagen are in purple. Offsets for acidified and unacidified excreta are labeled with “U” and “A” respectively.

On the basis of existing work, we had anticipated that raptor fecal matter would have slightly lower δ^13^C and slightly higher δ^15^N values than proteinaceous tissues in consumed prey (Reid et al. 2023). In general, estimated Δ^13^C_diet‐excreta_ for both Caspian and Rhett were similar to expectations and within the range previously reported for birds, as well as some mammals (Table 1; Figure 9). In contrast, Δ^15^N_diet‐excreta_ for the two raptors was very different from most previous reports for both birds and mammals (Figure 9). Carnivorous mammals tend to have quite low (very negative) Δ^15^N_diet‐feces_, and faunivorous seabirds also have negative Δ^15^N_diet‐feces_ (Table 1; Figure 9). Positive Δ^15^N_diet‐feces_ has only been previously reported for a few humans fed fish (Δ^15^Ndiet‐feces ranges from 0.2 to 1.0‰; Kuhnle et al. 2013) and red‐necked stints fed cereal‐ or fish‐based pellets (Δ^15^N_diet‐feces_ = 0.5 to 0.6‰; Kuwae et al. 2022; Table 1).

Acidifying excreta lowered δ^13^C and δ^15^N values (increasing the calculated diet‐excreta offset), which either suggests selective removal of some ^13^C‐ and ^15^N‐depleted acid‐ or water‐soluble compounds, or a possible negative impact of acidification that biased the isotopic composition of the samples, which we will discuss in more detail below (Jacob et al. 2005; Schlacher and Connolly 2014).

Variable inclusion of urine and urates in the excreta samples could have both elemental and isotopic impacts. Specifically, as reviewed below, we might expect inclusion of these compounds to result in larger weight %N (and consequently smaller atomic C:N), but it is a little more challenging to anticipate how urine or urates would affect excreta δ^13^C and δ^15^N values, if at all. Traditionally, researchers have considered that the majority of bird urine is uric acid (C_5_H_4_N_4_O_3_), although the exact composition is not known and may include ammonium urate (C_5_H_7_N_5_O_3_), struvite (H_18_MgNO_10_P), and other nitrogenous compounds that are yet to be identified (Crouch et al. 2020). All of these chemicals should have small C:N. Likewise, urate salts are rich in nitrogen and potassium, and therefore should also have large %N and small C:N (Casotti and Braun 2004). In agreement with these expectations, Bird et al. (2008) reported a C:N of 1.17 ± 1.13 for what they considered to be isolated uric acid that had been extracted from zebra finch (*Taeniopygia castanotis*) guano. Researchers have reported lower δ^15^N values in urine than feces for ungulates (Sutoh et al. 1987; Sponheimer et al. 2003) while the opposite has been found for humans consuming a variety of diets (Kuhnle et al. 2013). To our knowledge, carbon isotope values in both urine and feces from the same individuals have only been evaluated in two studies (both on humans), and opposite trends were reported: Kuhnle et al. (2013) found that urine has lower δ^13^C values than feces, while Kim et al. (2011) found lower δ^13^C values for feces than urine. Similarly, little work has been conducted on urates versus feces. Bird et al. (2008) reported lower δ^13^C and higher δ^15^N values in bulk guano compared to isolated uric acid for captive zebra finches and a variety of wild seabirds, while Mizutani and Wada (1988) reported that uric acid and bulk guano were isotopically indistinguishable for wild Adelie penguins ( *Pygoscelis adeliae* ) and black‐tailed gulls ( *Larus crassirostris* ).

**FIGURE 10:**
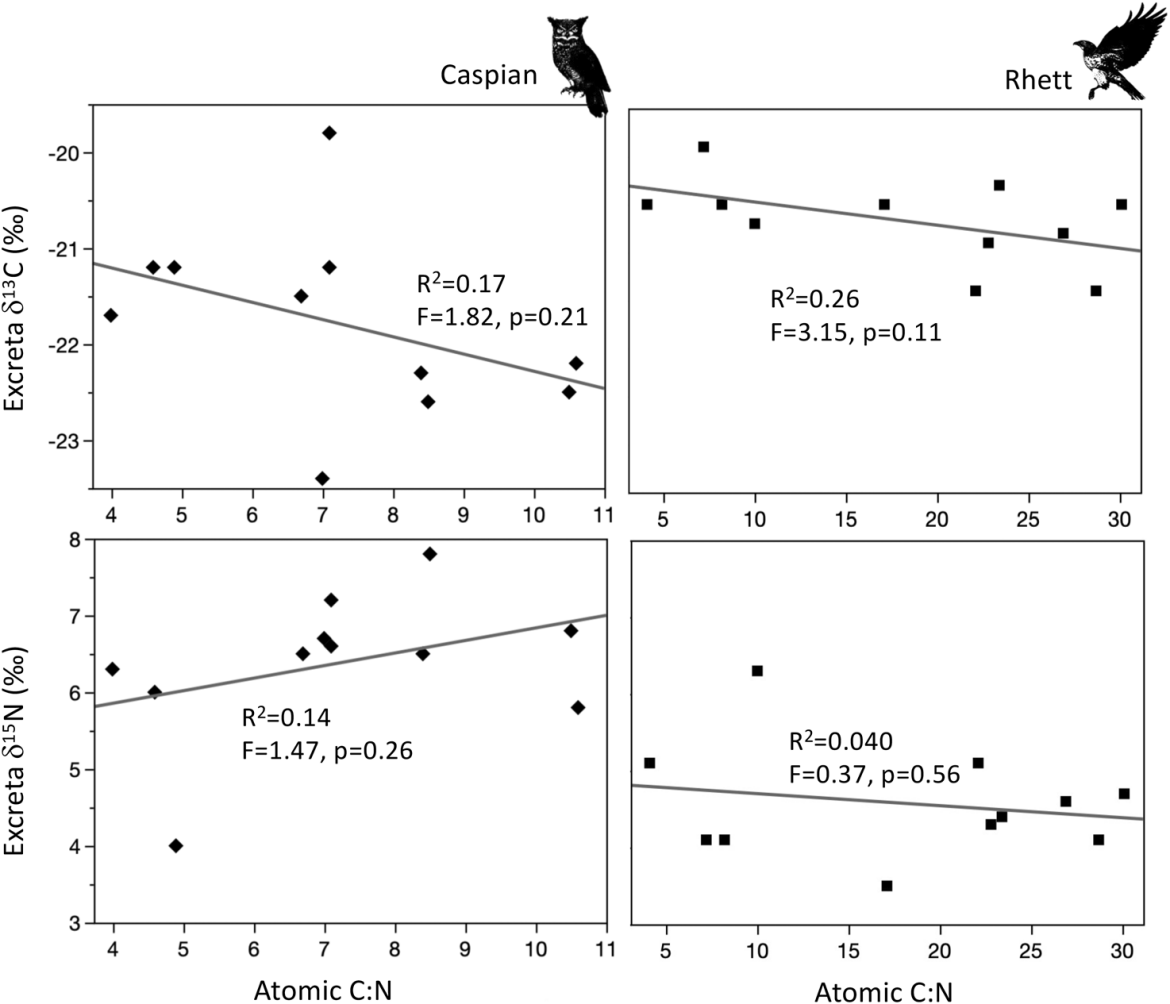
Bivariate plots and linear regression equations showing a lack of any relationships between atomic C:N and carbon and nitrogen isotope values for unacidified excreta.

Overall, the five “urate?” flecks we sampled had significantly lower atomic C:N than unacidified excreta, but only three of these had small atomic C:N (1.3–1.5) while the others had larger atomic C:N more similar to acidified excreta (Figure 6). These differences were due to relatively large weight %N for the three “urate?” samples with small C:N (Appendix 2). Relatively elevated %N suggests these samples were, indeed, urates (Casotti and Braun 2004). However, there were no clear visual or statistically significant differences in δ^13^C or δ^15^N among “urate?” flecks, untreated excreta, and acidified excreta (Figure 6). There were also no relationships between δ^13^C or δ^15^N and excreta atomic C:N for either Rhett or Caspian (Figure 10). These results align with some previous research (i.e., Mizutani and Wada 1988), and suggest that even if excreta did contain some urine or urate, these waste products might not have had a measurable impact on Δ_diet‐excreta_ calculations. We discuss this point in more detail in the next section.

As noted in the Introduction, other researchers working with birds have also not typically tried to isolate different waste products, and Δ^15^N_diet‐excreta_ for most species is negative (Table 1; Figure 9). Therefore, we feel an additional factor must be involved, perhaps one that is unique to birds of prey. One possibility is differences in digestive physiology between raptors and other kinds of birds. All bird digestive tracts are dominated by an esophagus, a muscular stomach, a short intestine, and a colon, but this apparent simplicity is deceptive. In fact, “When compared across species, the GI tract is the most anatomically diverse organ system” (Klasing 1999). Avian species vary tremendously in how their digesta are compartmentalized and how they function, including muscle action, digestive enzymes and gut microbes (Proszkowiec‐Weglarz 2022). Red‐tailed hawks and owls both have relatively short and simple digestive tracts compared to most birds (Duke 1997). Additionally, while many aquatic birds that eat animal matter have a separate chamber in their stomach, called the pyloric stomach, which collects bones and feathers (likely acting as a filter to prevent these materials from entering the intestines; Duke 1997), raptors eject undigestible material as a pellet. The pH of raptor stomachs is also relatively low (ca. 1.6 for hawks and 2.3 for owls; Duke et al. 1975). Freshly sacrificed fasting chickens, turkeys, ducks, pheasants, and pigeons also had a stomach pH around 2.0 (Proszkowiec‐Weglarz 2022). However, other portions of these species' digestive tracts had higher pH, and stomach pH is also likely higher when food is present (Proszkowiec‐Weglarz 2022). Active, wild Magellanic penguins ( *Spheniscus magellanicus* ) had variable stomach pH ranging from ca. 2 to 6 throughout the day (Peters 1997).

There are also considerable differences in digestive physiology between owls and hawks, which might help explain small differences in diet‐excreta offset between Caspian and Rhett (although we also cannot rule out that the observed differences may simply be due to the specific individuals included). In addition to differences in stomach pH, there may be differences in proteolytic activity and gut microbiota between distantly related owls and hawks (Houston and Duke 2007). Moreover, ceca (which are absent in hawks) are an important part of the digestive tract for groups of birds that retain this part of the large intestine. Ceca help with water absorption and recycling of nitrogen waste (Duke 1997). They are also a home for microorganisms that can digest high‐fiber foods like plant cellulose and starch that a bird may be otherwise poorly equipped to digest (Proszkowiec‐Weglarz 2022). Contribution of cecal microbes to owl digestion, and presence of sloughed microbes in Caspian's excreta could help explain some of the differences in excreta isotopic composition (and associated diet‐excreta offset) between the two birds. Avian gut microbiota are highly diverse and aid in a variety of functions ranging from assistance with digestion and nutrient absorption to detoxification of poisonous foods and immune function (Kohl 2012). While some microbes are common to all birds, there are likely some that are unique to specific avian taxa (Kohl 2012). The degree to which microbes might impact the isotopic composition of bird excreta is unknown. There is an isotopic influence of the gut microbiome on organism tissues, but it is complicated and has only been investigated in a handful of mammalian herbivores to date (Newsome et al. 2020; Besser et al. 2023).

Finally, there are differences in how owls and hawks consume their prey. Owls tend to swallow their meals whole, while falcons like to tear up their prey into little bits. Consequently, hawks may ingest fewer organs than owls, including stomachs and intestines. Houston and Duke (2007) hypothesized that a cecum might help owls digest plant matter in the guts of the prey they consume. We were not able to evaluate what was fed to the rats that were eaten by the birds in this study, but expect it was manufactured pellets. The contribution of food contained in rat guts should have been minor compared to rat tissues, but it could certainly have contributed to Caspian's diet and may be partially responsible for the small differences in diet‐excreta offset between the two birds.

Based on bioapatite, Δ^13^C_diet‐excreta_ ranged from 9.2 ± 1.3‰ to 10.0 ± 1.3‰ for Caspian and 8.7 ± 1.8‰ to 9.3 ± 1.8‰ for Rhett, depending on whether unacidified or acidified excreta were used in the calculation (Figure 8). Researchers do not typically consider diet‐excreta offset using bone (or tooth enamel) bioapatite. We are aware of just one study that has done so: Crowley et al. (2019) used isotope values from both bioapatite and collagen from isolated bone fragments recovered from fecal samples to estimate diet for wild jaguars ( *Panthera onca* ) in Belize. Nevertheless, bioapatite *is* used frequently to estimate diet, especially when coupled with bone collagen (which we discuss in more detail in section 4.5 below), and given the non‐destructive advantage of working with excreta, perhaps researchers will start pairing prey bioapatite and predator excreta more in the future.

Lastly, estimated Δ^87^Sr/^86^Sr_diet‐excreta_ for Caspian and Rhett (0.00086 ± 0.00045, and 0.0012 ± 0.00036, respectively) was similar to published values for guinea pigs ( *Cavia porcellus* ) and rats ( *Rattus rattus* ) fed insect pellets (Appendix 1). Guinea pigs fed meat pellets had much larger offsets, while rats fed meat pellets and guinea pigs and rats fed plant pellets both had smaller offsets. Pigs ( *Sus scrofa* ) fed diets with varying marine content also had much smaller offsets (both positive and negative). Although food is typically considered to be the primary source of ingested strontium, drinking water can also contribute (Watts and Howe 2010), and Weber et al. (2020) attributed differences in ^87^Sr/^86^Sr between diet and feces for rats and guinea pigs to the influence of drinking water. We did not analyze the water consumed by birds in our study, but the main drinking water source for the city of Cincinnati, including the zoo, is the Ohio River, which was previously reported to have an ^87^Sr/^86^Sr of 0.71145 in 2014 (Baumann and Crowley 2015) and 0.7116 in 1973 (Curtis and Stueber 1973). These ratios are higher than undigested bone ^87^Sr/^86^Sr and much higher than excreta ^87^Sr/^86^Sr. Other surface water sources in southwestern Ohio and northern Kentucky also had relatively elevated ^87^Sr/^86^Sr in 2014 (all > 0.70870; Baumann and Crowley 2015), which is the highest measured ^87^Sr/^86^Sr for any of the raptor excreta. Thus, we do not think that strontium from drinking water can explain low ^87^Sr/^86^Sr for excreta. We consider additional possible explanations for lower ^87^Sr/^86^Sr in excreta compared to diet in section 4.5 below.

### 4.2 Excreta Appearance

We encountered a large amount of variability in excreta appearance. Given Rhett and Caspian's consistent diet over the course of our study, we had not expected so much variability. As discussed in detail below, distinct isotopic and elemental signatures for excreta with different coloration and textures likely reflect variable contributions of waste products (feces, urine and urates), as well as the amount of time since food and water had been consumed.

The unusual “sulfur‐like” excreta for Caspian visually resembled the few “urate?” samples we processed. It did not have unusual isotopic values or atomic C:N prior to acid rinsing, but had considerably lower δ^13^C and smaller atomic C:N than other samples after acidification (Figure 5). Changes observed following acidification may reflect the removal of soluble urine. Soaking samples in acid and rinsing them with water repeatedly should have eluted and flushed at least some urine from the excreta (and possibly uric acid and urates, although these tend to be base soluble rather than acid soluble; Bird et al. 2008, Mizutani and Wada 1985). We observed isotopic shifts following acidification for most of the excreta. In general, acidified raptor excreta had lower δ^13^C values than unacidified samples (as expected if we flushed urine from the excreta). However, we also observed a considerable drop in δ^15^N values for acidified excreta (especially for Caspian), which is opposite the trend expected. We observed a slight increase in atomic C:N following acidification for some samples, but the overarching trend was for acidification to increase weight %N and reduce excreta C:N, including for the unusual “sulfur‐like” sample produced by Caspian (Appendix 3). Isotopic and elemental shifts may also have been due to the acid treatment itself (Jacob et al. 2005; Schlacher and Connolly 2014), which we will discuss in more detail in the next section. Thus, while it is almost certain that urine and urates were present in the excreta, their concentration was likely variable, and it is hard to account for their impact. Additional research on this topic is needed.

It is tempting to conclude that carbon from dissolved bone mineral is responsible for the very large atomic C:N observed for some unacidified excreta. Excreta with large C:N were nearly entirely ones with a “mud” texture (the exception being one “crumbles” sample produced by Rhett; Figure 5; Appendix 2). The majority of these samples were also produced by Rhett, who definitely digested some bone (only a few of her pellets contained enough bone to analyze). Smaller C:N (especially for “mud” samples) and slightly lower δ^13^C values following acidification would be consistent with excreta containing some dissolvable mineral. However, weight %C actually increased with acidification for most excreta textures (Appendix 3). It is only because weight %N increased relatively more than %C that acidified excreta had smaller atomic C:N than unacidified excreta. Dissolved bone carbonate may thus not be a satisfactory explanation for large atomic C:N in unacidified excreta.

Finally, variable excreta textures (and associated isotopic and elemental variability) may reflect the amount of time that excreta were in a bird's guts, as well as how long it had been since the bird ate a meal or consumed water. All of these factors would be expected to influence the relative contribution of recently consumed food, endogenous sloughed epithelial cells, mucus, digestive enzymes, and microbes to excreta (Klasing 1999). All birds excrete waste frequently (typically multiple times per hour) to avoid carrying unnecessary weight during flight. However, only one sample was collected from each bird's enclosure on any given day of the study, and we do not know when these particular samples would have been produced relative to when the birds consumed their meals. While this is unfortunate, to our knowledge, no researchers have previously evaluated temporal fluctuations in the isotopic or elemental composition of avian excreta. Instead, most researchers have analyzed bulk guano produced by one or more individuals over a 24‐h period (Table 1). A future study that can more fully account for potential temporal variability in excreta composition throughout the day would be beneficial.

### 4.3 Is It Wise to Acidify Excreta?

To date, acidifying excreta has not been a common practice, and it is not something we would have considered had we not encountered excreta with C:N far larger than what is typically reported for meat‐eating vertebrates, including faunivorous birds (Appendix 4). With the exception of slightly (ca. 1‰) lower δ^13^C values for the four excreta from Caspian with atomic C:*N* > 8, excreta with elevated atomic C:N did not have particularly unusual δ^13^C, δ^15^N, or ^87^Sr/^86^Sr, nor were there any relationships between atomic C:N and δ^13^C or δ^15^N for either bird (Figure 10; Appendix 2). We note that it is relatively uncommon for researchers to report excreta %C, %N, or C:N. Given that these data are generated along with isotopic data, and that they can be rather informative, we recommend including them in future work.

As noted above, acidification tended to lower excreta δ^13^C and δ^15^N values. Overall, the influence of acidification was small for most of the raptor excreta (< 1‰), but it was quite pronounced for a few samples (on the order of 3‰ for δ^15^N values for some of Caspian's excreta). These trends are consistent with what has been reported in previous studies on other types of biological tissues (Schlacher and Connolly 2014). It is difficult to disentangle the degree to which these isotopic shifts reflect removal of undesired material (such as mineral or non‐fecal waste products), or acidification biasing the isotopic composition of organics in the excreta. If acidification has negative impacts on organic components of a sample, then perhaps it is best not to do this step. Or perhaps it would be prudent to acidify an aliquot of sample for carbon analysis, but not for nitrogen analysis (Schlacher and Connolly 2014). However, if there are impacts of digested mineral on δ^15^N values, as suggested by Jacob et al. (2005), then acidification would be prudent for δ^15^N values as well. Schlacher and Connolly (2014) suggested that one can determine if a sample needs demineralizing by adding a few drops of acid to a sample and observing if the sample fizzes. However, we tried this “champagne test” and none of the excreta produced any bubbles, including those with large atomic C:N. Jacob et al. (2005) observed shifts in both δ^13^C and δ^15^N as well as N content of marine invertebrate and fish samples they expected to be carbonate‐free and noted that there may be issues associated with rinsing samples with water following acid treatment. These authors recommended adding 1 M acid drop by drop to a powdered sample until no more CO_2_ is released, and then not rinsing the samples. Yet, if samples do not effervesce at all, it is difficult to know if enough acid has been added. There may be alternative acids to HCl that would have less of an isotopic impact on organic components of samples (e.g., H_2_SO_4_ or even HF; Fernandes and Krull 2008), although both of these chemicals have considerable health and safety concerns. Clearly, sample treatment does matter, and more work on this is needed.

### 4.4 Tentative Recommendations to Account for Diet‐Excreta Offsets in Raptors

We acknowledge the very limited scope of this preliminary study. More work is needed to confirm if the patterns we observed in Δ^13^C_diet‐excreta_ and Δ^15^N_diet‐excreta_ for Caspian and Rhett are consistent in other eagle owls and red‐tailed hawks, let alone present in other birds of prey. As such, we are very cautious with any recommendations and leave it up to the reader to decide the degree to which they might want to account for diet‐excreta offset in their own work. Despite physiological and behavioral differences, Δ^13^C_diet‐excreta_ for Rhett and Caspian was actually quite similar if we considered diet to have included both bone and muscle for Rhett (who did, indeed, digest a good deal of bone) but exclusively muscle for Caspian (0.7 vs. 0.9‰ for unacidified excreta and 1.3 vs. 1.7‰ for acidified excreta; Figure 7). Given these broad similarities for the two birds, we tentatively suggest a Δ^13^C_diet‐excreta_ of ca. 1‰ when working with unacidified excreta and 1.5‰ when working with acidified excreta. Diet‐excreta offset for nitrogen was considerably smaller for Caspian than Rhett no matter how we evaluated diet when working with unacidified excreta. Yet values became much more similar for acidified excreta: 0.7‰ for Rhett if we consider both bone and muscle contributed to diet vs. 0.9‰ for Caspian if only muscle contributed (Figure 7). As mentioned above, it is possible that acidifying samples had an undesirable influence on excreta δ^15^N (and the apparent similarities in Δ^15^N_diet‐excreta_ for the two birds are simply coincidence). However, as we also noted above, we think it is equally likely that some urine and urate were removed from the samples during acidification and subsequent rinsing, which would make these values more valid than those for unacidified excreta. We therefore tentatively suggest a Δ^15^N_diet‐excreta_ of 0.5–1‰ for both birds.

Carbon diet‐excreta offset based on bone apatite was also quite similar for the two birds (but slightly larger for Caspian; Figure 8). Acidifying excreta increased estimated offsets by ca. 0.6 to 0.8‰. Overall, we feel an offset of 9–10‰ is reasonable for both birds. Lastly, we tentatively recommend a diet‐excreta offset of 0.001 for ^87^Sr/^86^Sr, which is roughly the average of the offsets calculated for the two birds (0.00086 for Rhett and 0.0012 for Caspian; Figure 8).

### 4.5 Influence of Digestion on the Composition of Prey Tissues

We observed small isotopic and elemental shifts in the composition of some rat tissues following digestion (Figures 2, 3, 4). Additional research exploring the potential factor(s) responsible for both elemental and isotopic shifts in consumed prey tissues is needed. Nevertheless, we believe the patterns we have observed are robust.

**FIGURE 11:**
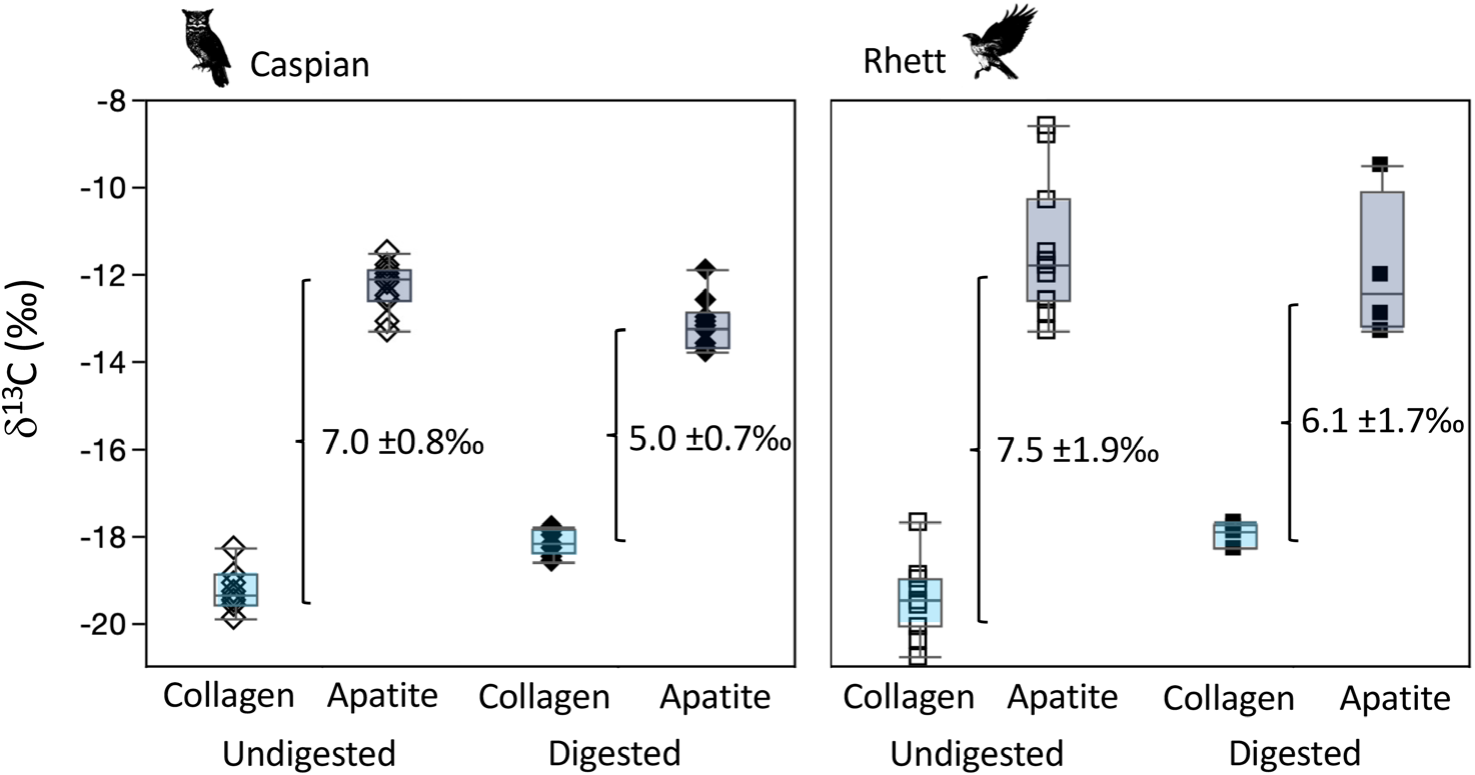
Box plots and summary estimates (mean ± 1*σ*) for δ^13^C offset between collagen and bioapatite both for undigested rodent bone (removed from rat cadavers prior to feeding Caspian the eagle owl and Rhett the red‐tailed hawk), and digested rodent bones dissected from pellets regurgitated by the two birds.

Fur keratin weight %C and weight %N declined with digestion for both Rhett and Caspian (Figure 2). However, digestion did not impact keratin atomic C:N, δ^13^C or δ^15^N values, which is reassuring for any researchers who may want to analyze fur dissected from raptor pellets. There also appeared to be no influence of digestion on collagen δ^15^N values or bioapatite δ^18^O values (although we note that δ^18^O data were highly variable overall; Figures 3 and 4). However, there were significant impacts of digestion on both collagen and bioapatite δ^13^C values as well as strontium isotopes (Figures 3 and 4), and these shifts have implications for data interpretations based on digested bone. Although shifts in collagen and bioapatite δ^13^C values were small, they were in opposite directions. Consequently, the collagen‐apatite spacing for digested bone was 1.5 to 2‰ smaller than for undigested bone for both Caspian and Rhett (Figure 11). This reduction in collagen‐apatite spacing would impact our interpretation of the diet consumed by the predated rats. Faunivores tend to have smaller collagen‐apatite spacing than omnivores, and herbivores tend to have larger spacing than omnivores (Bocherens et al. 2017; Lee‐Thorp et al. 1989). We might therefore conclude that the rats had a diet richer in animal matter than they did in reality. The reduction in carbonate‐collagen offset would also impact models that rely on the offset in δ^13^C between collagen and apatite to estimate the δ^13^C value of dietary protein (Pestle et al. 2015).

We suggest that the negative shift in bioapatite δ^13^C values reflects selective dissolution of mineral rich in ^13^C during digestion. We would expect dissolution of mineral to have been more pronounced for Rhett, who digested more bone, yet the decrease in δ^13^C is only significant for Caspian (Figure 4). This may be because bioapatite data for rats fed to Rhett were much more variable.

Removal of lipids is the most likely explanation for the observed positive shifts in collagen δ^13^C following digestion; this would also be expected to result in smaller weight %C and atomic C:N (Jim et al. 2004), both of which were observed. Despite repeated sonication in petroleum ether, which should have been effective at removing lipids (Dobush et al. 1985), all but one of the undigested bones had atomic C:N > 3.6, which is typically considered to be the cutoff for well‐preserved, pure collagen (reviewed in Van Klinken 1999). However, after initially analyzing these samples, we re‐sonicated them several additional times with petroleum ether and observed no liberated lipids or changes in either δ^13^C values or atomic C:N. We therefore do not think lipids are a satisfactory explanation for the low δ^13^C values and elevated atomic C:N. The four samples with the largest atomic C:N (< 4.2) had the lowest δ^13^C values (ca. −20 to −21‰; Table 1) but excluding these four samples, there was no relationship between δ^13^C and atomic C:N for undigested collagen (*R* ^2^ = 0.10 F_1,17_ = 0.18, *p* = 0.20), and there were still clear differences in both δ^13^C values and atomic C:N between digested and undigested bone collagen for both birds (Figure 3). It is possible that digestion removed lipids that were not otherwise extractable from bone, or had some impact on the relative efficiency and efficacy of subsequent chemical treatment. Yet neither the presence of lipids nor lipid extraction should have influenced δ^15^N isotopes (Dobush et al. 1985), and there was a very slight (but insignificant) drop in collagen δ^15^N following digestion for Rhett. Selective hydrolysis of some amino acids is another possible explanation for isotopic shifts in collagen following digestion. This would be expected to impact both δ^13^C and δ^15^N values, and depending on which amino acids were affected, could have had a variable impact on weight %C, weight %N, and atomic C:N (Hare et al. 1991). This is a possibility that needs to be explored further.

A drop in ^87^Sr/^86^Sr following digestion is somewhat perplexing given the mass of strontium and previous research that has reported negligible fractionation of ^87^Sr/^86^Sr with biological uptake (Capo et al. 1998; Flockhart et al. 2015). Nevertheless, our data demonstrate that heavy ^87^Sr was preferentially removed from bone during digestion, and it was not excreted (excreta had ^87^Sr/^86^Sr that was comparable to digested bone; Table 3). It is possible that the birds sequestered calcium (and strontium, which readily substitutes for calcium) to build eggshells (Blum et al. 2001). However, our study took place outside of the normal egg‐laying season (Spring), and to our knowledge, neither Rhett nor Caspian laid any eggs during the sample collection period. Perhaps ^87^Sr was preferentially incorporated into bird osseous tissues, which we were not able to sample. Blum et al. (2001) found that bones of warblers ( *Dendroica caerulescens* ) had lower ^87^Sr/^86^Sr than foods the birds consumed during the summer, but the authors attributed this to the birds consuming an isotopically distinct winter diet that also contributed some to bone ^87^Sr/^86^Sr.

While a shift in ^87^Sr/^86^Sr of 0.0005 to 0.001 following digestion may seem very small to researchers accustomed to working with other isotope systems, the observed reduction in rat bone ^87^Sr/^86^Sr following digestion by both Caspian and Rhett is large enough to impact interpretations of the bioavailable Sr consumed by rats. One might interpret an ^87^Sr/^86^Sr of 0.708 to 0.709 as marine carbonates, while a ratio higher than this would typically be interpreted to indicate some contribution of siliciclastic sediments or silica‐rich igneous rocks (Capo et al. 1998). Consequently, using rodent bones accumulated by owls to establish expected bioavailable ^87^Sr/^86^Sr for a study area, as is sometimes done in archaeological studies (e.g., Ezzo et al. 1997), could be problematic.

Digestion does leave telltale signs on bones (Terry 2007; Terry et al. 2018), and we recommend that researchers examine bones with a microscope, or possibly a hand lens, to check for pitting and etching. It would likely be best to avoid specimens that show signs of having been digested, but if this is not possible, one may be able to account for (and possibly correct for) the isotopic influence of digestion.

## 5 Conclusions

In summary, we set out to achieve two goals in this study: (1) Conduct a pilot analysis of diet‐excreta offset for a Eurasian eagle owl and a red‐tailed hawk; and (2) Evaluate the degree to which digestion impacts the isotopic composition of consumed prey tissues. Overall, we found small diet‐excreta offset (+0 to 2‰) when using carbon and nitrogen isotopes in consumed rat proteinaceous tissues to estimate diet, but patterns were somewhat different for the two birds. This likely reflects differences in how the two species consume and digest their prey, but may also be due to the specific individuals included. Diet‐excreta offset based on rodent bioapatite was +9 to 10‰ for carbon and +0.001 for strontium for both birds. We note that this level of variability is on par with the kinds of error associated with typical assumptions of diet‐tissue offset. However, we are also very aware of the limitations of our dataset; we stress that our study was relatively small in scope and more work is needed. An expanded study that evaluates how consistent the isotopic trends observed in this study are for other individual eagle owls and red‐tailed hawks, as well as other raptor species, is warranted. In particular, a study that can more fully control for all inputs (such as the isotopic composition of imbibed water and dietary details for consumed prey), establish the isotopic composition of different waste products, and investigate the factors responsible for variability in excreta appearance and composition (including, but not limited to, amount of water and food intake, and time since last meal) would also be extremely helpful. It could be especially illuminating to investigate diet‐excreta offset for a vulture, which digests all of the bone it consumes. It could also be informative to study an owl within the Tytonidae, which is phylogenetically distinct from strigid owls, or a species that specializes in avian prey that requires active pursuit (e.g., peregrine falcons, *Falco peregrinus* ). Avian specialists tend to have shorter intestines than generalist species that perch and ambush prey (like those included in the present study), and consequently have lower digestive efficiency, requiring more food or higher quality food than generalists to meet their metabolic needs (Barton and Houston 1993). This could conceivably affect the isotopic composition of their excreta, which would, in turn, impact diet‐excreta offset.

Rather unexpectedly, we found that digestion does impact δ^13^C values in both bone collagen and bone apatite, as well as ^87^Sr/^86^Sr in bone apatite. What goes in is isotopically distinct from what comes out. The mechanisms behind these shifts are somewhat elusive, and again, we stress that more work is needed. Nevertheless, the shifts seem to be robust, and they are large enough to potentially impact data interpretations. It would be useful to evaluate the degree to which enamel is impacted by digestion. One would hope that it is more resistant to alteration than bone, but this would be very good to confirm in a future study.

## Author Contributions

**Brooke Erin Crowley:** conceptualization (equal), formal analysis (lead), investigation (equal), project administration (lead), visualization (lead), writing – original draft (lead), writing – review and editing (lead). **Madison Laurel Greenwood:** conceptualization (equal), formal analysis (equal), funding acquisition (lead), investigation (equal), writing – review and editing (supporting). **Rachel Elizabeth Brown Reid:** conceptualization (supporting), visualization (supporting), writing – review and editing (supporting).

## Conflicts of Interest

The authors declare no conflicts of interest.

## Data Availability

Details for all individual samples, including raw isotope data, are provided in Appendix 2. These data have also been archived with the Dryad data repository (https://doi.org/10.5061/dryad.ksn02v7ft).

## Appendix 1 Published Δ^87^Sr/^86^Sr_diet‐feces_

**Table:**

Common name	Scientific name	Diet	*N*	Average offset	± 1*σ*	Source	Notes
Pig	*Sus scrofa*	100% soy protein	4	0.000015	0.014	Lewis et al. (2017)	Uncertainty is 2*σ* in ppm (as reported by authors)
Pig	*Sus scrofa*	82.5% soy; 12.5% fish meal	4	‐0.00004	0.014	Lewis et al. (2017)	Uncertainty is 2*σ* in ppm (as reported by authors)
Pig	*Sus scrofa*	75% soy; 25% fish meal	4	‐0.000022	0.019	Lewis et al. (2017)	Uncertainty is 2*σ* in ppm (as reported by authors)
Pig	*Sus scrofa*	50% soy; 50% fish meal	4	0.000051	0.016	Lewis et al. (2017)	Uncertainty is 2*σ* in ppm (as reported by authors)
Pig	*Sus scrofa*	100% fish meal	4	‐0.000002	0.017	Lewis et al. (2017)	Uncertainty is 2*σ* in ppm (as reported by authors)
Norway rat	*Rattus norvegicus*	Plant pellet	1	‐0.00021		Weber et al. (2020)	Data extracted from figure and offset estimated
Norway rat	*Rattus norvegicus*	Insect pellet	1	0.00065		Weber et al. (2020)	Data extracted from figure and offset estimated
Norway rat	*Rattus norvegicus*	Meat pellet	1	0.00043		Weber et al. (2020)	Data extracted from figure and offset estimated
Guinea pig	*Cavia porcellus*	Plant pellet	1	‐0.00065		Weber et al. (2020)	Data extracted from figure and offset estimated
Guinea pig	*Cavia porcellus*	Insect pellet	1	0.0009		Weber et al. (2020)	Data extracted from figure and offset estimated
Guinea pig	*Cavia porcellus*	Meat pellet	1	0.0029		Weber et al. (2020)	Data extracted from figure and offset estimated
Jaguar	*Panthera onca*	Wild game	10	0.0022	0.0067	Crowley et al. (2019)	Diet estimated based on bone fragments recovered from feces

## Appendix 2 Details and raw isotope data for each sample included in this study

**Table:**

Individual Bird	Date collected	Tissue	Day of Study	ẟ^13^C_collagen_ (‰)	Weight %C	Amp 44	ẟ^15^N (‰)	Weight %N	Amp 28	Atomic C:N	ẟ^13^C_bioapatite_ (‰)	ẟ^18^O (‰)	^87^Sr/^86^Sr	Sr concentration (accounting for sample mass)	Notes
Caspian	25‐10‐2018	Predigested Bone	1	−19.5	43.0	6434	5.4	14.2	4051		−13.3	−6.8	0.70865		Statistical outlier for δ^18^O and Sr concentration
Caspian	25‐10‐2018	Predigested Muscle	1	−20.8	51.0	7772	6.6	14.2	4356						
Caspian	25‐10‐2018	Predigested Hair	1	−19.2	50.2	12630	6.5	15.6	6691						
Caspian	26‐10‐2018	Post‐digested Hair	1	−19.3	47.7	8910	5.6	14.2	5350						
Caspian	26‐10‐2018	Post‐digested Excreta	1	−21.2	17.8	11242	4.0	4.2	2836				0.70807		Dark brown "crumbles"
Caspian	26‐10‐2018	Post‐digested Acidified Excreta	1	−22.3	57.3	19587	4.4	8.9	5705						Dark brown "crumbles"
Caspian	26‐10‐2019	Post‐digested Bone	1	−18.6	41.4	7109	5.4	14.8	4888		−13.2	−4.1	0.70804	65.89	
Caspian	26‐10‐2018	Predigested Bone	2	−18.3	47.8	10686	6.1	15.8	5999		−12.6	−5.3	0.70894		
Caspian	26‐10‐2018	Predigested Muscle	2	−21.0	51.4	7272	5.8	15.3	4324						
Caspian	26‐10‐2018	Predigested Hair	2	−19.2	46.2	11429	5.9	14.4	6103	3.7					
Caspian	27‐10‐2018	Post‐digested Bone	2	−18.3	41.0	7200	5.7	15.4	5221	3.1	−13.3	−4.1	0.70809		
Caspian	27‐10‐2018	Post‐digested Hair	2	−19.2	44.9	8271	5.8	14.0	5145	3.8					
Caspian	27‐10‐2018	Post‐digested Excreta	2	−21.7	15.3	11063	6.3	4.4	2349	4.0			0.70835		Dark brown "crumbles"
Caspian	27‐10‐2018	Post‐digested Acidified Excreta	2	−22.7	58.7	22616	4.3	8.3	6033	8.3					Dark brown "crumbles"
Caspian	27‐10‐2018	Predigested Bone	3	−19.6	43.3	6951	6.0	13.7	4188	3.7	−13.1	−3.6	0.70879		
Caspian	27‐10‐2018	Predigested Muscle	3	−20.6	48.6	6928	6.8	14.2	4032	4.0					
Caspian	27‐10‐2018	Predigested Hair	3	−18.9	48.5	12265	6.5	15.3	6606	3.7					
Caspian	28‐10‐2018	Post‐digested Bone	3	−18.2	39.9	6324	4.8	15.5	4680	3.0	−13.0	−4.9	0.70808		
Caspian	28‐10‐2018	Post‐digested Hair	3	−19.2	41.9	8348	6.3	13.8	5280	3.5					
Caspian	28‐10‐2018	Post‐digested Excreta	3	−21.2	18.9	12121	6.0	4.8	2871	4.6			0.70833		
Caspian	28‐10‐2018	Post‐digested Acidified Excreta	3	‐22.7	58.5	19377	4.8	8.3	5113	8.3					Dark brown "crumbles"
Caspian	28‐10‐2018	Post‐digested Urate?	3	−21.3	31.8	21068	7.0	27.6	35379	1.3					Dark brown "crumbles"
Caspian	03‐07‐2019	Predigested Bone	4	−19.1	49.2	11747	5.7	15.3	6241	3.8	−11.9	−4.8	0.70997	212.96	
Caspian	03‐07‐2019	Predigested Hair	4	−19.2	49.2	9535	6.0	14.9	5623	3.9					
Caspian	03‐07‐2019	Predigested Muscle	4	−20.8	52.9	11146	6.3	14.4	5926	4.3					
Caspian	04‐07‐2019	Post‐digested Bone	4	−18.3	45.0	6683	5.9	14.9	5141	3.5	−11.9	−3.7	0.70841	61.75	Very little bone. Looks like one individual
Caspian	04‐07‐2019	Post‐digested Excreta	4	−19.8	54.1	6390	6.6	8.9	26361	7.1			0.70878	4.01	“shiny” glassy bits
Caspian	04‐07‐2019	Post‐digested Acidified Excreta	1	−19.8	61.3	19755	6.3	8.8	5314	8.1					“shiny” glassy bits
Caspian	04‐07‐2019	Post‐digested Urate?	1	−22.14	20.0	7664	3.6	3.7	2512	6.3					White‐yellow flecks
Caspian	04‐07‐2019	Post‐digested Hair	4	−19.2	42.9	6846	6.4	13.1	4544	3.8					
Caspian	04‐07‐2019	Predigested Bone	5	−19.4	47.3	10224	5.7	13.9	5137	4.0	−12.1	−4.0	0.70970	138.82	
Caspian	04‐07‐2019	Predigested Hair	5	−19.2	47.7	7504	5.9	14.4	5297	3.9					
Caspian	04‐07‐2019	Predigested Muscle	5	−20.9	52.4	7279	6.1	14.5	4566	4.2					
Caspian	05‐07‐2019	Post‐digested Bone	5	−17.9	46.3	6337	5.8	15.5	4858	3.5	−13.1	−4.4	0.70806	53.56	Pellet contained two feathers (small and colorful; not like eagle owl feathers)
Caspian	05‐07‐2019	Post‐digested Excreta	5	−22.3	44.9	1641	6.5	6.2	17846	8.4			0.70851	40.92	"Sulfur‐like" yellow‐gray
Caspian	05‐07‐2019	Post‐digested Acidified Excreta	5	−24.3	39.8	12762	6.1	31.6	18900	1.5					"Sulfur‐like" yellow‐gray
Caspian	05‐07‐2019	Post‐digested Urate?	5	−22.3	44.9	1641	6.5	6.2	17846	8.4					White/tan flecks
Caspian	05‐07‐2019	Post‐digested Hair	5	−19.3	45.8	7067	6.0	13.1	4410	4.1					
Caspian	05‐07‐2019	Predigested Bone	6	−19.9	51.6	12996	5.7	13.1	5677	4.6	−11.5	−4.1	0.70973	129.21	
Caspian	05‐07‐2019	Predigested Hair	6	−19.0	47.7	8991	6.2	14.5	5338	3.9					
Caspian	05‐07‐2019	Predigested Muscle	6	−21.1	67.3	7071	6.1	18.2	4451	4.3					Weight %C and %N are both statistical outliers for muscle.
Caspian	06‐07‐2019	Post‐digested Excreta	6	−21.2	53.7	6303	7.2	8.9	27269	7.1			0.70855	3.12	“shiny” glassy bits
Caspian	06‐07‐2019	Post‐digested Excreta	6	−21.2	45.1	2036	5.4	3.2	10087	16.3					Tan "mud"
Caspian	06‐07‐2019	Post‐digested Acidified Excreta	6	−21.2	60.0	20640	6.5	8.8	5662	8.0					“shiny” glassy bits
Caspian	06‐07‐2019	Post‐digested Acidified Excreta	6	−22.0	57.0	19538	4.8	10.2	6565	6.6					Tan "mud"
Caspian	06‐07‐2019	Predigested Bone	7	−19.6	49.1	11193	5.1	14.4	5572	4.0	−12.0	−4.2	0.70964	93.01	
Caspian	06‐07‐2019	Predigested Hair	7	−19.3	50.0	7346	6.2	13.4	4438	4.4					
Caspian	06‐07‐2019	Predigested Muscle	7	−20.6	50.5	12258	6.5	14.3	5965	4.1					
Caspian	07‐07‐2019	Post‐digested Bone	7	−17.8	47.8	6992	6.2	16.2	5524	3.5	−13.6	−4.2	0.70809	49.81	Three rats in pellet (12 incisors)
Caspian	07‐07‐2019	Post‐digested Excreta	7	−22.2	45.8	2381	5.8	5.0	15024	10.6			0.70850	66.38	Dark brown "crumbles"
Caspian	07‐07‐2019	Post‐digested Hair	7	−19.0	48.2	11743	6.4	14.7	6122	3.8					
Caspian	08‐07‐2019	Predigested Bone	8	−18.9	47.9	11561	6.0	14.9	6193	3.7	−12.2	−2.6	0.70929	98.83	
Caspian	08‐07‐2019	Predigested Hair	8	−19.2	48.2	9624	6.3	14.5	5648	3.9					
Caspian	08‐07‐2019	Predigested Muscle	8	−19.2	39.7	8094	6.7	11.4	4530	4.1					δ^13^C for this sample is a statistical outlier for muscle.
Caspian	09‐07‐2019	Post‐digested Excreta	8	−23.4	49.8	4521	6.7	8.3	25211	7.0			0.70894	23.47	Dark brown "crumbles"
Caspian	09‐07‐2019	Post‐digested Acidified Excreta	8	−23.0	48.0	17719	5.2	9.1	6297	6.1					Dark brown "crumbles"
Caspian	09‐07‐2019	Post‐digested Urate?	8	−22.4	33.4	13448	6.49	30.2	22671	1.3					
Caspian	09‐07‐2019	Predigested Hair	9	−19.0	50.6	10746	6.1	15.0	6190	4.0					
Caspian	09‐07‐2019	Predigested Bone	9	−19.3	49.0	7629	5.4	13.5	4954	4.2	−11.9	−4.5	0.70969	113.29	
Caspian	09‐07‐2019	Predigested Muscle	9	−20.6	53.3	11110	6.7	13.9	5638	4.5					
Caspian	10‐07‐2019	Post‐digested Bone	9	−17.8	51.1	6983	5.7	16.7	5335	3.6	−13.7	−3.2	0.70814	59.04	Three rats in pellet (12 incisors)
Caspian	10‐07‐2019	Post‐digested Excreta	9	−22.6	46.6	2961	7.8	6.4	18903	8.5			0.70848	57.71	Dark brown "crumbles"
Caspian	10‐07‐2019	Post‐digested Acidified Excreta	9	−22.9	63.9	21957	4.7	7.7	4989	9.7					
Caspian	10‐07‐2019	Post‐digested Hair	9	−19.2	45.7	7577	6.0	12.9	4686	4.1					
Caspian	10‐07‐2019	Predigested Bone	9	−18.9	49.0	11445	6.0	15.2	6057	3.8	−11.8	−3.5	0.70979	126.84	
Caspian	10‐07‐2019	Predigested Hair	10	−19.1	47.6	10596	5.8	14.5	6271	3.8					
Caspian	10‐07‐2019	Predigested Muscle	10	−20.5	51.6	9893	6.7	13.5	5047	4.5					
Caspian	11‐07‐2019	Post‐digested Bone	10	−18.5	47.1	6681	5.2	15.2	5044	3.6	−13.7	−4.0	0.70815	69.00	Two rats in pellet; analyzed bioapatite for both
Caspian	11‐07‐2019	Post‐digested Bone	10							0.0	−13.8	−2.9	0.70817	59.02	Two rats in pellet; analyzed bioapatite for both
Caspian	11‐07‐2019	Post‐digested Excreta	10	−21.5	52.4	5642	6.5	9.2	27324	6.7			0.70890	6.22	“shiny” glassy bits
Caspian	11‐07‐2019	Post‐digested Excreta	10	−22.3	45.1	1887	7.9	3.3	9319	16.0					Tan "mud"
Caspian	11‐07‐2019	Post‐digested Acidified Excreta	10	−21.9	58.7	21716	6.2	8.7	6130	7.8					“shiny” glassy bits
Caspian	11‐07‐2019	Post‐digested Acidified Excreta	10	−22.3	55.0	17701	5.5	11.2	6736	5.7					Tan "mud"
Caspian	11‐07‐2019	Post‐digested Hair	10	−19.4	45.3	6704	5.6	13.5	4356	3.9					
Caspian	11‐07‐2019	Predigested Bone	11	−19.6	49.9	11347	5.5	14.8	5776	3.9	−12.5	−4.4	0.70935	87.19	
Caspian	11‐07‐2019	Predigested Hair	11	−19.3	49.1	7542	6.0	14.6	5081	3.9					
Caspian	11‐07‐2019	Predigested Muscle	11	−20.8	51.1	7687	5.4	13.2	4638	4.5					
Caspian	12‐07‐2019	Post‐digested Bone	11	−18.0	45.8	6813	5.9	15.5	5386	3.4	−12.6	−4.2	0.70825	59.02	Likely 1 rat (only one partial incisor)
Caspian	12‐07‐2019	Post‐digested Excreta	11	−22.5	45.2	1811	6.8	5.0	14254	10.5			0.70846	50.87	Tan "mud"
Caspian	12‐07‐2019	Post‐digested Acidified Excreta	11	−22.9	53.0	17981	5.5	14.4	9164	4.3					Tan "mud"
Caspian	12‐07‐2019	Post‐digested Hair	11	−18.8	41.3	6648	5.8	12.6	4704	3.8					
Rhett	25‐10‐2018	Predigested Hair	1	−18.8	47.4	11294	5.8	15.1	6120	3.7					
Rhett	25‐10‐2018	Predigested Bone	1	−19.0	42.8	6850	6.0	14.3	4353	3.5	−13.3	−5.9	0.70938		
Rhett	25‐10‐2018	Predigested Muscle	1	−20.6	51.1	7602	6.4	13.2	4320	4.5					
Rhett	26‐10‐2018	Post‐digested Hair	1	−18.9	42.7	8790	6.2	13.9	5475	3.6					
Rhett	26‐10‐2018	Post‐digested Excreta	1	−19.9	12.8	2016	4.1	2.1	5238	7.2			0.70839		Dark brown "crumbles"
Rhett	26‐10‐2018	Post‐digested Acidified Excreta	1	−20.9	52.5	20703	5.03	8.9	6771	6.9					Dark brown "crumbles"
Rhett	26‐10‐2018	Predigested Hair	2	−17.7	48.0	11777	5.5	14.8	6201	3.8					
Rhett	26‐10‐2018	Predigested Muscle	2	−19.2	51.5	7185	5.6	14.0	3879	4.3					
Rhett	26‐10‐2018	Predigested Bone	2	−18.9	51.0	10916	6.1	14.5	5296	4.1	−10.3	−4.1	0.71002		
Rhett	27‐10‐2018	Post‐digested Hair	2	−18.7	42.3	9376	5.9	13.8	5888	3.6					
Rhett	27‐10‐2018	Post‐digested Excreta	2	−20.5	16.8	6166	4.1	2.4	2656	8.2			0.70839		Tan "mud"
Rhett	27‐10‐2018	Post‐digested Acidified Excreta	2	−21.1	52.3	21500	4.6	7.8	6139	7.8					Tan "mud"
Rhett	27‐10‐2018	Predigested Bone	3	−19.2	42.7	6850	5.5	14.5	4465	3.4	−12.9	−4.7	0.70913		
Rhett	27‐10‐2018	Predigested Hair	3	−19.0	48.2	12115	5.9	15.3	6654	3.7					
Rhett	27‐10‐2018	Predigested Muscle	3	−20.2	49.2	7232	5.7	14.5	4261	4.0					
Rhett	28‐10‐2018	Post‐digested Hair	3	−19.3	42.2	8930	5.4	12.0	4864	4.1					
Rhett	28‐10‐2018	Post‐digested Bone	3	−18.3	35.1	6345	5.2	13.2	4569	3.1	−12.9	−4.5	0.70815		
Rhett	28‐10‐2018	Post‐digested Excreta	3	−20.5	18.9	13807	5.1	5.4	2845	4.1			0.70846		Tan "mud"
Rhett	28‐10‐2018	Post‐digested Acidified Excreta	3	−21.3	48.8	19050	4.4	7.3	5453	7.8					Tan "mud"
Rhett	28‐10‐2018	Post‐digested Urate?	3	−20.0	29.3	9783	6.3	23.0	14435	1.5					
Rhett	03‐07‐2019	Predigested Bone	4	−17.7	47.9	7275	4.5	14.7	5072	3.8	−8.6	−3.6	0.71021	101.69	Predigested rat isotopically distinct from the one analyzed in the pellet.
Rhett	03‐07‐2019	Predigested Hair	4	−18.2	48.4	10075	5.7	14.7	5956	3.9					
Rhett	03‐07‐2019	Predigested Muscle	4	−20.5	51.9	7863	5.8	14.5	5115	4.2					
Rhett	04‐07‐2019	Post‐digested Bone	4	−17.9	47.1	6466	5.7	15.9	5074	3.5	−13.3	−3.0	0.70820	67.09	
Rhett	04‐07‐2019	Post‐digested Excreta	4	−20.3	46.5	2220	4.4	2.3	6393	23.4			0.70878	79.61	Tan "mud"
Rhett	04‐07‐2019	Post‐digested Acidified Excreta	4	−20.5	52.3	19583	4.3	8.1	5828	7.5					Tan "mud"
Rhett	04‐07‐2019	Post‐digested Hair	4	−19.4	51.0	7682	5.3	14.4	5065	4.1					
Rhett	04‐07‐2019	Predigested Hair	5	−19.0	46.9	6815	5.2	14.3	4863	3.8					
Rhett	04‐07‐2019	Predigested Bone	5	−19.6	49.6	11609	5.4	14.1	5698	4.1	−8.8	−3.5	0.70972	97.56	
Rhett	04‐07‐2019	Predigested Muscle	5	−21.4	51.2	7577	6.0	13.9	4811	4.3					
Rhett	05‐07‐2019	Post‐digested Bone	5	−17.8	46.3	6244	4.9	14.4	4465	3.8	−9.5	−6.8	0.70887	62.38	
Rhett	05‐07‐2019	Post‐digested Excreta	5	−20.5	46.3	1714	4.7	1.8	4852	30.1			0.70894	54.13	Tan "mud"
Rhett	05‐07‐2019	Post‐digested Acidified Excreta	5	−21.0	57.4	23075	4.8	7.9	6129	8.5					Tan "mud"
Rhett	05‐07‐2019	Post‐digested Hair	5	−18.8	49.9	13212	5.4	15.1	6901	3.9					
Rhett	05‐07‐2019	Predigested Bone	6	−20.8	44.8	7183	5.3	10.7	3732	4.9	−11.5	−3.3	0.71005	123.63	
Rhett	05‐07‐2019	Predigested Hair	6	−19.3	50.0	9824	6.1	15.3	5854	3.8					
Rhett	05‐07‐2019	Predigested Muscle	6	−21.2	51.2	7467	6.4	14.2	4821	4.2					
Rhett	06‐07‐2019	Post‐digested Bone	6	−17.7	47.0	6842	4.9	15.0	5100	3.7					
Rhett	06‐07‐2019	Post‐digested Excreta	6	−20.9	46.7	2579	4.3	2.4	6873	22.8			0.70900	42.82	Tan "mud"
Rhett	06‐07‐2019	Post‐digested Acidified Excreta	6	−21.8	48.2	19094	4.7	6.1	4537	9.3					Tan "mud"
Rhett	06‐07‐2019	Post‐digested Hair	6	−19.6	47.7	7041	5.7	13.8	4753	4.0					
Rhett	06‐07‐2019	Predigested Bone	7	−19.2	49.0	10974	6.2	14.0	5355	4.1	−11.7	−3.4	0.70975	97.98	
Rhett	06‐07‐2019	Predigested Hair	7	−19.2	49.7	9232	5.9	15.1	5483	3.8					
Rhett	06‐07‐2019	Predigested Muscle	7	−21.1	53.4	7877	5.8	14.9	5134	4.2					
Rhett	07‐07‐2019	Post‐digested Bone	7	−18.3	46.9	7108	5.4	15.2	5381	3.6	−12.01	−4.63	0.70821	54.49	
Rhett	07‐07‐2019	Post‐digested Excreta	7	−20.7	51.4	5227	6.3	6.0	17267	10.1			0.70859	39.17	Dark brown "crumbles"
Rhett	07‐07‐2019	Post‐digested Hair	7	−19.6	49.9	7389	5.8	14.3	4942	4.1					
Rhett	08‐07‐2019	Predigested Bone	8	−20.4	46.6	7428	5.4	12.3	4287	4.4	−12.0	−3.5	0.70941	107.88	
Rhett	08‐07‐2019	Predigested Hair	8	−19.1	48.2	9275	5.8	14.4	5670	3.9					
Rhett	08‐07‐2019	Predigested Muscle	8	−20.9	51.8	10697	6.2	13.2	5565	4.6					
Rhett	09‐07‐2019	Post‐digested Excreta	8	−21.4	45.6	1815	4.1	1.9	4881	28.7			0.70876	63.89	Tan "mud"; no pellet
Rhett	09‐07‐2019	Post‐digested Acidified Excreta	8	−21.8	50.8	19628	4.5	7.4	5432	8.0					Tan "mud"
Rhett	09‐07‐2019	Post‐digested Hair	8	−19.0	45.7	6826	5.9	14.1	4903	3.8					
Rhett	09‐07‐2019	Predigested Bone	9	−19.5	40.7	6023	5.3	11.4	3693	4.2	−12.6	−3.0	0.70934	100.55	
Rhett	09‐07‐2019	Predigested Hair	9	−19.1	49.2	9048	5.8	15.0	5398	3.8					
Rhett	09‐07‐2019	Predigested Muscle	9	−20.6	51.4	13992	6.1	13.7	6399	4.4					
Rhett	10‐07‐2019	Post‐digested Excreta	9	−21.4	46.0	1970	5.1	2.4	6791	22.1			0.70857	71.39	Tan "mud"
Rhett	10‐07‐2019	Post‐digested Acidified Excreta	9	−22.0	50.9	19761	5.3	7.6	5603	7.9					Tan "mud"
Rhett	10‐07‐2019	Post‐digested Hair	9	−19.0	48.0	7021	5.5	13.9	4723	4.0					
Rhett	10‐07‐2019	Predigested Bone	10	−20.1	34.2	5377	5.3	8.9	3044	4.5	−12.0	−4.8	0.70963	99.45	
Rhett	10‐07‐2019	Predigested Hair	10	−19.4	43.4	7047	6.4	13.3	4725	3.8					
Rhett	10‐07‐2019	Predigested Muscle	10	−20.6	52.0	10491	6.7	13.3	5232	4.6					
Rhett	11‐07‐2019	Post‐digested Excreta	10	−20.8	46.0	1816	4.6	2.0	5792	26.9			0.70906	47.40	Tan "mud"
Rhett	11‐07‐2019	Post‐digested Acidified Excreta	10	−21.4	57.6	23417	4.5	7.8	6102	8.6					Tan "mud"
Rhett	11‐07‐2019	Post‐digested Hair	10	−19.4	49.2	7813	4.9	14.5	5368	4.0					
Rhett	11‐07‐2019	Predigested Bone	11	−19.5	49.3	7072	5.8	13.8	4601	4.2	−11.8	−3.8	0.70997	124.28	
Rhett	11‐07‐2019	Predigested Hair	11	−19.3	47.8	9191	5.7	14.4	5400	3.9					
Rhett	11‐07‐2019	Predigested Muscle	11	−18.5	46.6	7742	6.0	14.9	4841	3.6					
Rhett	12‐07‐2019	Post‐digested Excreta	11	−20.5	48.2	3443	3.5	3.3	9404	17.1			0.70891	64.24	Dark brown "crumbles"
Rhett	12‐07‐2019	Post‐digested Acidified Excreta	11	−20.6	59.4	22215	3.9	6.3	4494	11.0					Dark brown "crumbles"
Rhett	12‐07‐2019	Post‐digested Hair	11	−18.9	47.6	11776	5.1	14.6	6154	3.8					

*Note*: Blank cells indicate no data. Carbon and oxygen isotope data are presented relative to the VPDB scale. Additional details, including (including weight %C:N, strontium blanks and measured Sr concentration without accounting for sample mass), are available in dataset archived with Dryad (https://doi.org/10.5061/dryad.ksn02v7ft).

## Appendix 3 Summary Data and Statistical Comparisons of Isotopic and Elemental Data Among Excreta Textures for Each Bird Before and After Acid Treatment

**Table:**

Individual	Acidified?	Texture	δ^13^C (‰)			δ^15^N (‰)			Weight %C			Weight %N			Atomic C:N			^87^Sr/^86^Sr			Sr Concentration (μg/g sample)		
			*N*	Mean	± 1*σ*	*N*	Mean	± 1*σ*	*N*	Mean	± 1*σ*	*N*	Mean	± 1*σ*	*N*	Mean	± 1*σ*	*N*	Mean	± 1*σ*	*N*	Mean	± 1*σ*
Caspian	No	“Shiny”	3	−20.8	0.9	3	6.8	0.4	3	53.4	0.9	3	9.0^a^	0.20	3	7.0	0.2	3	0.70874	0.00018	3	4.5	1.6
		“Crumbles”	6	−21.5	1.4	6	5.6	1.5	6	35.1	17.0	6	6.7^a^	1.7	6	7.6	2.1	6	0.70845	0.00029	3	49.2	22.7
		“mud”	3	−22.0	0.7	3	6.7	1.3	3	45.2	0.1	3	3.9^a^	1.0	3	14.3	3.2	1			1	50.9	
		“Sulfur‐like”	1	−21.3		1	6.8		1	44.9		1	6.2^a^		1	6.6		1			1	40.9	
				χ^2^ = 1.51; df = 3; *p* = 0.66			χ^2^ = 2.59; df = 3; *p* = 0.46			χ^2^ = 6.60; df = 3; *p* = 0.086			χ^2^ = 8.18; df = 3; ***p* = 0.042**			χ^2^ = 7.10; df = 3; *p* = 0.069			χ^2^=3.88; df=3; *p*=0.27			χ^2^=5.22; df=3; *p*=0.16	
				Levene *F* = 3.83; df = 2, 9; *p* = 0.063			Levene *F* = 1.93; df = 2, 9; *p* = 0.20			Levene *F* = 39.05; df = 2, 9; ***p* < 0.0001**			Levene *F* = 4.09; df = 2, 9; *p* = 0.060			Levene *F* = 3.13; df = 2, 9; ***p* = 0.036**			Levene *F* = 0.30; df = 1, 7; *p* = 0.60			Levene *F* = 10.28; df = 1, 4; ***p* = 0.033**	
	Yes	“Shiny”	5	−22.7^a^	1.1	5	4.7^a^	0.2	5	60.0	1.3	5	8.8	0.03	5	8.0	0.2						
		“Crumbles”	3	−21^a^	0.3	3	6.3^a^	0.4	3	55.7	5.1	3	8.6	0.4	3	8.0	1.3						
		“Mud”	3	−22.4^a^	0.5	3	5.3^a^	0.4	3	55.0	2.0	3	11.9	2.2	3	5.5	1.2						
		“Sulfur‐like”	1	−24.3^a^		1	6.1^a^		1	39.9		1	31.6		1	1.5							
				χ^2^ = 8.20; df = 3; ***p* = 0.042**			χ^2^ = 9.16; df = 3; **p = 0.027**			χ^2^ = 7.03; df = 3; *p* = 0.071			χ^2^ = 7.27; df = 3; *p* = 0.064			χ^2^ = 6.9; df = 3; *p* = 0.076							
				Levene *F* = 3.97; df = 2, 8; *p* = 0.063			Levene *F* = 1.32; df = 2, 8; *p* = 0.32			Levene *F* = 2.59; df = 2, 8; *p* = 0.20			Levene *F* = 9.92; df = 2, 8; ***p* = 0.0091**			Levene *F* = 1.6; df = 2, 8; *p* = 0.26							
Rhett	No	“Crumbles”	3	−20.4	0.4	3	4.6	1.5	3	37.5	21.4	3	3.8	2.0	3	11.4	5.1	3	0.70863	0.00026	3	51.7	17.7
		“Mud”	8	−20.7	0.3	8	4.8	0.9	8	39.1	13.1	8	2.6	1.2	8	18.6	11.1	8	0.70874	0.00025	8	59.9	14.3
				χ^2^ = 0.88; df = 1; *p* = 0.35			χ^2^ = 0.68; df = 1; *p* = 0.41			χ^2^ = 0.67; df = 1; *p* = 0.41			χ^2^ = 1.50; df = 1; *p* = 0.22			χ^2^ = 0.68; df = 1; *p* = 0.41			χ^2^ = 0.38; df = 1; *p* = 0.54			χ^2^=0.44; df=1; p=0.51	
				Levene *F* = 0.15; df = 1, 9; *p* = 0.71			Levene *F* = 1.81; df = 1, 9; *p* = 0.21			Levene *F* = 1.62; df = 1, 9; *p* = 0.23			Levene *F* = 1.52; df = 1, 9; *p* = 0.25			Levene *F* = 6.27; df = 1, 9; ***p* = 0.034**			Levene *F* = 0.46; df = 1; *p* = 0.83			Levene *F* = 0.029; df = 1; *p* = 0.87	
	Yes	“Crumbles”	2	−20.8	0.2	2	4.5	0.8	2	56.0	4.9	2	7.6	1.9	2	9.0	2.9						
		“Mud”	8	−21.4	0.5	8	4.7	0.3	8	52.3	3.5	8	7.5	0.7	8	8.2	0.6						
				χ^2^ = 2.47; df = 1; *p* = 0.17			χ^2^ = 0.068; df = 1; *p* = 0.79			χ^2^ = 2.45; df = 1; *p* = 0.12			χ^2^ = 0.068; df = 1; *p* = 0.79			χ^2^ = 0.0; df = 1; *p* = 1.0							
				Levene *F* = 1.34; df = 1, 8; *p* = 0.28			Levene *F* = 5.1; df = 1, 8; *p* = 0.054			Levene *F* = 0.27; df = 1, 8; *p* = 0.62			Levene *F* = 7.27; df = 1, 8; ***p* = 0.027**			Levene *F* = 50.23; df = 1, 8; ***p* = 0.016**							

*Note:* Significant results are presented in bold and marginally significant results are underlined. Textures that share a superscript letter within each comparison are statistically indistinguishable using Steel–Dwass all pairs tests.

## Appendix 4 Published C:N for Various Organisms From a Literature Search

**Table:**

Organism type	Common name	Species	Simplified type of consumer	Min C/N	Max C/N	Mean C/N	SD	N	Acidified?	Sample treatment	Notes	Citation
Bird	Zebra finch	*Taeniopygia castanotis*	Herbivore	2.9	4.2	3.6	0.7	4	No	Homogenized	Birds fed a mixed C3/C4 seed diet for the first 24 days of the study. This is the average of samples collected on days 7, 14, 21 and 24 of the study	Bird et al. (2008)
Bird	Zebra finch	*Taeniopygia castanotis*	Herbivore	3.2	4.5	3.7	0.5	7	No	Homogenized	Birds fed A C3 seed diet after 24 days. This is average data for samples collected on days 28, 29, 30, 32, 36, and 39 of the study. Data for day 26 of the study not included as it seemed the animals hadn't yet equilibrated to the new diet.	Bird et al. (2008)
Bird	Adelie Penguin	*Pygoscelis adeliae*	Faunivore			1.8	—	4	No	Samples stored frozen; then dried and homogenized	Diet values are estimated	Mizutani and Wada (1988)
Bird	Black‐legged kittiwake	*Rissa tridactyla*	Faunivore	1.6	1.9	1.7	0.1	5	No	Homogenized		Bird et al. (2008)
Bird	Black‐tailed Gull	*Larus crassirostris*	Faunivore			2.2	—	4	No	Homogenized	Diet values are estimated	Mizutani and Wada (1988)
Bird	Great skua	*Stercorarius skua*	Faunivore	1.7	2.1	2.0	0.2	5	No	Homogenized		Bird et al. (2008)
Bird	Manx shearwater	*Puffinus puffinus*	Faunivore	1.1	1.3	1.2	0.1	5	No	Homogenized		Bird et al. (2008)
Bird	Northern Fulmar	*Fulmarus glacialis*	Faunivore	1.1	1.4	1.2	0.1	5	No	Homogenized		Bird et al. (2008)
Bird	Northern gannet	*Morus bassanus*	Faunivore	1.2	2.8	1.6	0.7	5	No	Homogenized		Bird et al. (2008)
Bird	Swallow	*Hirundo rustica*	Faunivore			3.1		1	No	Homogenized		Bird et al. (2008)
Bird	Chicken	*Gallus gallus*	Trophic omnivore	—	—	11.0	—	1	No	Homogenized		Bertholt et al. (2021)
Bird	Herring gull	*Larus argentatu*	Trophic omnivore	5.1	11.7	6.9	4.2	3	No	Homogenized		Bird et al. (2008)
Bird	Red‐necked stint	*Calidris ruficollis*	Trophic omnivore			5.6	1.8	394	Yes	Samples homogenized and treated with 1M HCl; no other details provided		Kuwae et al. (2022)
Bird	Red‐necked stint	*Calidris ruficollis*	Trophic omnivore			6.9	2.6	77	Yes	Samples homogenized and treated with 1M HCl; no other details provided		Kuwae et al. (2022)
Bird	Red‐necked stint	*Calidris ruficollis*	Trophic omnivore			3.1	0.5	20	Yes	Samples homogenized and treated with 1M HCl; no other details provided		Kuwae et al. (2022)
Mammal	Cow	*Bos taurus*	Herbivore	—	—	17.5	0.1	—	No	Samples treated with K2SO4. No other details provided		Wang et al. (2018)
Mammal	Chimpanzee	*Pan troglodytes*	Herbivore	8.4	29	—	—	—	No	Homogenized	Infant; only min and max C:N reported	Bădescu et al. (2017)
Mammal	Chimpanzee	*Pan troglodytes*	Herbivore	12	27.6	—	—	—	No	Homogenized	Juvenile; only min and max C:N reported	Bădescu et al. (2017)
Mammal	Chimpanzee	*Pan troglodytes*	Herbivore	11.1	29.7	—	—	—	No	Homogenized	Mother; only min and max C:N reported	Bădescu et al. (2017)
Mammal	Etawa goat	*Capra hircus*	Herbivore	—	—	13.0	—	—	No	Homogenized		Saputra et al. (2023)
Mammal	François' langur	*Trachypithecus francoisi*	Herbivore	7	14.8	—	—	—	No	Homogenized	Infant; only min and max C:N reported	Reitsema (2012)
Mammal	François' langur	*Trachypithecus francoisi*	Herbivore	10.7	17.9	—	—	—	No	Homogenized	Mother; only min and max C:N reported	Reitsema (2012)
Mammal	Frugivorous bats	Various	Herbivore	—	—	25.0	13.0	9	No	Homogenized	Tested a subset; no difference in isotope values for acid and non‐acid treated samples	Gallant et al. (2021)
Mammal	Rabbit	*Oryctolagus cuniculus*	Herbivore	—	—	28.6	—	1	No	Homogenized		Bertholt et al. (2021)
Mammal	Sheep	*Ovis aries*	Herbivore	—	—	14.7	0.1	—	No	Samples treated with K2SO4. No other details provided	Samples treated with K2SO5	Wang et al. (2018)
Mammal	Bobcat	*Lynx rufus*	Faunivore	—	—	6.7	1.1	57	Yes	Samples soaked in 0.1 N HCl followed by Mili‐Q water; then homogenized		Reid (2015)
Mammal	Insectivorous bats	Various	Faunivore	—	—	4.7	1.1	17	No	Tested a subset; no difference in isotope values for acid and non‐acid treated samples		Gallant et al. (2021)
Mammal	Jaguar	*Panthera onca*	Faunivore	4.2	7.3	5.6	0.8	10	No	Homogenized	Samples ranged in age from < 24 h up to 10 days old	Crowley et al. (2019)
Mammal	Sanguinivorous bats	Various	Faunivore	—	—	5.7	1.2	2	No	Tested a subset; no difference in isotope values for acid and non‐acid treated samples. Samples Homogenized		Gallant et al. (2021)
Mammal	African grass rat	*Arvicanthis nilotocus*	Trophic omnivore	—	—	21.1	9.6	12	No	Homogenized		Bergstrom (2013)
Mammal	Coyote	*Canis latrans*	Trophic omnivore	—	—	8.6	2.3	28	Yes	Samples soaked in 0.1 N HCl followed by Mili‐Q water; then homogenized		Reid (2015)
Mammal	Fringe‐tailed gerbil	*Gerbilliscus robustus*	Trophic omnivore	—	—	6.8	2.2	9	No	Homogenized		Bergstrom (2013)
Mammal	Gray fox	*Urocyon cinereoargenteus*	Trophic omnivore	—	—	12.1	5.6	25	Yes	Samples soaked in 0.1 N HCl followed by Mili‐Q water; then homogenized		Reid (2015)
Mammal	Hinde's rock rat	*Aethomys hindei*	Trophic omnivore	—	—	9.0	4.3	5	No	Homogenized		Bergstrom (2013)
Mammal	Mearn's pouched mouse	*Saccostomus mearnsi*	Trophic omnivore	—	—	10.2	3.5	46	No	Homogenized		Bergstrom (2013)
Mammal	Mouse	*Mus* sp.	Trophic omnivore	—	—	10.9	1.7	13	No	Homogenized		Bergstrom (2013)
Mammal	Natal multimammate mouse	*Mastomys natalensis*	Trophic omnivore	—	—	11.2	3.6	4	No	Homogenized		Bergstrom (2013)
Mammal	Pig	*Sus domesticus*	Trophic omnivore	—	—	14.0	—	1	No	Homogenized		Bertholt et al. (2021)
Mammal	Spiny mouse	*Acomys* sp.	Trophic omnivore	—	—	11.1	5.9	14	No	Homogenized		Bergstrom (2013)
Terrestrial arthropood	Grasshopper	*Melanoplus femurrubrum*	Herbivore	—	—	11.6	0.2	—	No	Homogenized		Hawlena and Schmitz (2010)
Terrestrial arthropood	Grasshopper	*Melanoplus femurrubrum*	Herbivore	—	—	11.1	0.2	—	No	Homogenized		Hawlena and Schmitz (2010)
Marine invertebrate	Red sea urchin	*Mesocentrotus franciscanus*	Herbivore	—	—	23.0	—	—		Methodology not provided	Raw data not provided; estimated from figure	Dethier et al. (2019)
Marine invertebrate	Krill	*Euphusiacea* sp.	Trophic omnivore	—	—	5.3	—	—	No	Homogenized		Small et al. (1983)
Marine invertebrate	Mussel	*Aulacomya ater*	Trophic omnivore	—	—	4.9	0.2	11	No	Homogenized		Stuart et al. (1982)
Marine invertebrate	Salp	*Salpa fusiformis*	Trophic omnivore	—	—	7.4	—	—	No	Homogenized		Small et al. (1983)
Marine invertebrate	Tuna crab	*Pleuroncodes planipes*	Trophic omnivore	—	—	6.2	—	—	No	Homogenized		Small et al. (1983)
Marine invertebrate	Large zooplankton	Various	NA—mixed sample	—	—	6.5	—	—	No	Homogenized		Small et al. (1983)
Marine invertebrate	Mixed large and small zooplankton	Various	NA—mixed sample	—	—	7.1	—	—	No	Homogenized		Small et al. (1983)
Marine invertebrate	Small zooplankton	Various	NA—mixed sample	—	—	6.4	—	—	No	Homogenized		Small et al. (1983)
Marine invertebrate	Zooplankton	Various	NA—mixed sample	—	—	6.5	0.4	4	Yes	Homogenized	Population sample; thousands of individuals present	Altabet and Small (1990)
Marine invertebrate	Zooplankton	Various	NA—mixed sample	—	—	6.1	0.8	6	Yes	Homogenized	Population sample; thousands of individuals present	Altabet and Small (1990)
Marine invertebrate	Zooplankton	Various	NA—mixed sample	—	—	5.5	0.6	8	Yes	Homogenized	Population sample; thousands of individuals present	Altabet and Small (1990)
Marine invertebrate	Zooplankton	Various	NA—mixed sample	—	—	5.9	0.5	5	Yes	Homogenized	Population sample; thousands of individuals present	Altabet and Small (1990)

*Note:* Most authors do not clarify if C:N is weight % or atomic. However, the difference between these two values is small (the conversion is atomic C:N = 1.167 × weight %C:N), so this would only have a minor influence on reported C:N.
